# Simple Non-Equilibrium Atmospheric Plasma Post-Treatment Strategy for Surface Coating of Digital Light Processed 3D-Printed Vanillin-Based Schiff-Base Thermosets

**DOI:** 10.1021/acsapm.3c01632

**Published:** 2023-09-25

**Authors:** Anna Liguori, Huan Xu, Doli Hazarika, Minna Hakkarainen

**Affiliations:** † Department of Fibre and Polymer Technology, KTH Royal Institute of Technology, Teknikringen 58, 100 44 Stockholm, Sweden; ‡ School of Materials Science and Physics, China University of Mining and Technology, 221116 Xuzhou, China

**Keywords:** digital light processing 3D printing, biobased thermosets, plasma post-treatment, lignosulfonate coating, UV blocking, antioxidant properties

## Abstract

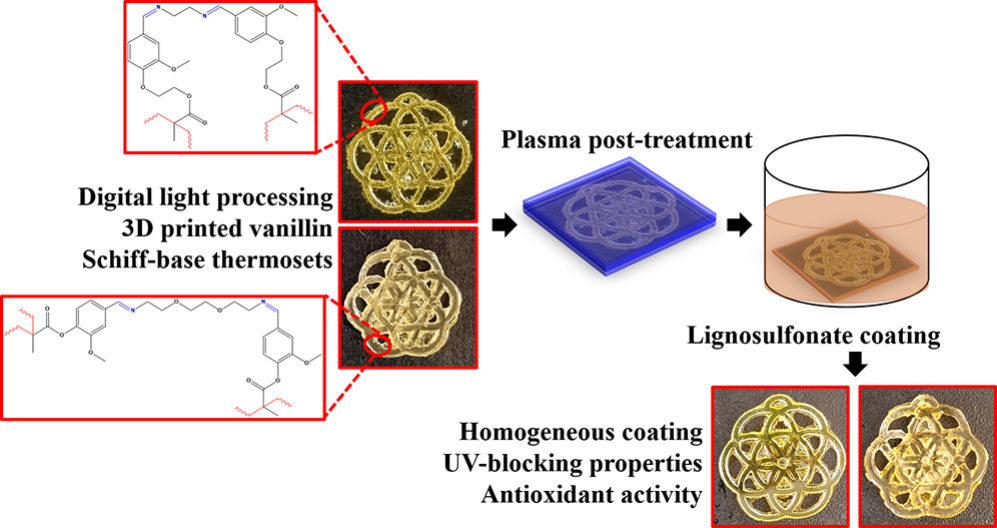

A simple non-equilibrium atmospheric plasma post-treatment strategy was developed for the surface coating of three-dimensional (3D) structures produced by digital light processing 3D printing. The influence of non-equilibrium atmospheric plasma on the chemical and physical properties of vanillin-derived Schiff-base thermosets and the dip-coating process was investigated and compared to the influence of traditional post-treatment with UV-light. As a comparison, thermosets without post-treatment were also subjected to the coating procedure. The results document that UV post-treatment can induce the completion of the curing of the printed thermosets if complete curing is not reached during printing. Conversely, the plasma post-treatment does not contribute to the curing of the thermoset but causes some opening of the imine bonds and the regeneration of aldehyde functions. As a consequence, no great differences are observed between the not post-treated and plasma post-treated samples in terms of mechanical, thermal, and solvent-resistant properties. In contrast to the UV post-treatment, the plasma post-treatment of the thermosets induces a noticeable increase of the thermoset hydrophilicity ascribed to the reformation of amines on the thermoset surface. The successful coating process and the greatest uniformity of the lignosulfonate coating on the surface of plasma post-treated samples are considered to be due to the presence of these amines and aldehydes. The investigation of the UV shielding properties and antioxidant activities documents the increase of both properties with the increasing amount and uniformity of the formed coating. Interestingly, evident antioxidant properties are also shown by the noncoated thermosets, which are deduced to their chemical structures.

## Introduction

For the development of tridimensional thermosets, digital light processing (DLP) three-dimensional (3D) printing is a widely employed vat photopolymerization process, which by diverting light from the source (typically with a wavelength in the range of 380–405 nm), enables the development of complex geometries starting from photoactive resins through a layer-by-layer curing approach.^1,2^ Despite the increasing interest toward this technology for the printing of photopolymerizable resins, the coupling of DLP with post-processing coating procedures to modify the surface properties of the printed objects is so far hardly investigated. A suitable methodology for the post-treatment of DLP 3D-printed thermosets would enable the introduction of functional groups on the surface, paving the way to the employment and exploitation of DLP 3D-printed materials also in fields where the surface properties play a critical role, i.e., optical transmission, biomedical applications, adhesion, antifriction, etc.

A few works proposed the DLP 3D printing of thermosets from commercial resins, followed by a dip-coating procedure. Alam et al. used DLP 3D printing for the obtainment of contact lenses starting from a commercial resin. To reach the desired optical transmittance, the printed lenses were dip-coated in the liquid resin for 1 min and cured under a UV lamp for 30 s.^3^ A similar approach was used for the realization of a protective coating onto the surface of DLP 3D-printed denture polymers obtained from a commercial resin; specifically, the uncured resin was coated on the surface of the printed objects to fill the staircase-like morphology and light postcured for 15 min.^4^ However, in these works, the coatings were realized from the same resin employed for the printing, and therefore, no new functionalities were added to the printed materials. More interestingly, Zhao et al. reported the DLP 3D printing of silicone-based elastomers followed by a dip-coating step in a 1:4 ethyl cyanoacrylate adhesive–trimethylpentane dispersion with the aim to favor the bonding of the silicon elastomer with a poly(vinyl alcohol) conductive hydrogel.^5^

In the context of the post-treatment strategies, the DLP 3D printing is often followed by approaches like polishing to flatten the surface of the printed objects;^6,7^ UV post-treatment to eliminate the influence of the photo-cross-linking gradient and to reach the completion of the curing after the printing;^8–17^ thermal postcuring generally adopted in the presence of a thermally curable component in the resin^18,19^ or to improve the thermal stability and mechanical properties of the printed structures.^20^ Although UV post-treatment is commonly employed after printing, the effects of this process are often not investigated in detail. In this frame, Lebedevaite et al. analyzed the mechanical properties of thermosets, obtained from commercially available biobased resins, after the UV post-treatment; the authors highlighted a modification of the mechanical properties suggesting the formation of more cross-linked structures.^21^ Wu et al. documented the shape distortion and reduction of dimension accuracy after the UV post-treatment due to the generation of an inhomogeneous stress field inside the printed structure.^22^ To the best of our knowledge, a systematic investigation of the effects of UV post-treatment, including the study of the resulting chemical and physical properties of the printed polymers, is still missing. Moreover, the role of the thermoset’s structure in the resulting effects of the UV post-treatment is not clearly established.

Among the techniques used for the surface modification of polymeric materials, non-equilibrium atmospheric pressure plasma is acknowledged as a versatile, solvent-free, and environmentally friendly approach. In this configuration, the plasma is generated by using plasma sources operating at atmospheric pressure, and the non-equilibrium conditions established in the plasma discharge enable the treatment of the surface of thermosensitive substrates without affecting their bulk properties.^23–25^ This aspect represents an intrinsic advantage of the plasma technology with respect to wet chemical treatments, which can negatively affect the structural and chemical integrity of the polymeric substrates.^26^ Another advantage of this technology relies on the versatility of atmospheric plasma sources, which can be properly designed to treat complex geometries. Non-equilibrium atmospheric pressure plasma has been employed over the years for numerous processes, such as the deposition of organic and organic–inorganic coatings;^27–30^ the grafting of biomolecules;^31,32^ and polymers,^33^ the chemical modification of the surface of the polymeric substrate.^34–37^ However, the coupling of this technology with the DLP 3D printing for the chemical modification of the surface of the polymeric prints is an unexplored area.

Correctly designed post-treatment of the surface of DLP-printed devices could offer a route for the creation of favorable interfacial interactions to facilitate the coating of the 3D devices with functional surface layers. We present a systematic investigation of the effects of non-equilibrium atmospheric pressure plasma on the chemical, mechanical, and thermal properties of biobased Schiff-base thermosets obtained by DLP 3D printing. As a comparison, the influence of commonly employed post-treatment with UV-light is investigated. We especially focus on the potential of these post-treatment strategies as tools for the creation of favorable interactions to enable coating of the thermosets with functional molecules, such as lignosulfonate, and the properties, such as UV shielding and antioxidant activity, induced by the coating. It is envisioned that the final thermoset properties and successful coating process depend on the selected post-treatment strategy and thereby the induced interactions between the surface and coating.

## Experimental Section

### Materials

Vanillin (V) (99%), ethylene carbonate (EC) (98%), potassium carbonate (K_2_CO_3_) (≥99%), *N*,*N*-dimethylformamide (DMF) (≥99%), methacrylic anhydride (MAA) (94%), ethylenediamine (ED) (≥99%), 2,2′-(ethylenedioxy)bis(ethylamine) (Dom) (>97%), phenylbis(2,4,6-trimethylbenzoyl)phosphine oxide (BAPO) (97%), and deuterium dimethyl sulfoxide (DMSO-*d*_6_) (99.9% atom % D) were purchased from Sigma-Aldrich and used without any additional purification. 4-(Dimethylamino)pyridine (DMAP) (≥99.0%, Fluka), magnesium sulfate (99%, VWR), sodium bicarbonate (≥99%, Merck), sodium hydroxide (NaOH) (≥99%, Fisher Scientific), ethyl acetate (EtOAc) (≥99%, VWR), dichloromethane (DCM) (≥99%, Fisher Scientific), hydrochloric acid (HCl) (37%), and acetone (≥99.5%, VWR) were all used as received. Sodium lignosulfonate for the coating of the thermosets and 2,2-diphenyl-1-picrylhydrazyl (DPPH°) for the antioxidant assay were acquired from Tokyo Chemical Industry Co., Ltd.

### Synthesis of the Resins

R1 and R2 were obtained by employing a triple-step and a double-step procedure, respectively. R1 was synthesized according to the protocol reported in our previous work.^9^ The procedure included the synthesis of extended vanillin (ExtV), its methacrylation, and the Schiff-base reaction with ethylene diamine (ED). R2 was produced by performing the methacrylation of vanillin (V) and the Schiff-base reaction with 2,2′-(ethylenedioxy)bis(ethylamine) (Dom).^11^ The protocol for the synthesis of both resins is briefly reported in the following.

ExtV was obtained by adding 10 g of V (65.72 mmol), 6.38 g of EC (72.45 mmol), and 11 g of K_2_CO_3_ (79.59 mmol) in a 250 mL round-bottom flask in the presence of 100 mL of DMF. The reaction mixture was kept under reflux in a N_2_ atmosphere at 110 °C for 12 h. Then, it was cooled down to room temperature, diluted with water (150 mL), and poured into EtOAc (150 mL). The aqueous phase was separated and extracted with EtOAc (2 × 100 mL). The organic phases were mixed, washed with water (2 × 200 mL), dried over magnesium sulfate, concentrated under reduced pressure, and further dried in a vacuum oven at 60 °C for 12 h.

M-ExtV was synthesized by mixing 10.00 g of ExtV (51.02 mmol), 8.58 g of MAA (55.66 mmol), and 38.00 mg of DMAP (0.32 mmol) in a 100 mL round-bottom flask. The reaction was carried out at 60 °C for 24 h under reflux and N_2_ flow. The resulting product was diluted with DCM (300 mL) and consequentially washed with a saturated aqueous solution of sodium bicarbonate (2 × 300 mL), 0.5 M NaOH (2 × 300 mL), 1 M NaOH (2 × 300 mL), and distilled water (150 mL). The organic phase was dried over magnesium sulfate, concentrated at reduced pressure, and dried under vacuum at 30 °C for 2 days. A similar procedure was employed for the production of methacrylated vanillin (MV): 10.13 g of V (66.58 mmol), 11.30 g of MAA (73.30 mmol), and 0.056 g of DMAP (0.46 mmol) were added to a 250 mL round-bottom flask with a magnetic stir bar under reflux and N_2_ flow. The reaction was again carried out at 60 °C for 24 h, and the resulting mixture was washed and dried according to the procedure used for M-ExtV.

Finally, the two resins were obtained by inducing a Schiff-base reaction between the aldehyde functions of M-ExtV and MV with the amine groups of ED and Dom, respectively. In particular, a solution of M-ExtV (12.00 g, 45.40 mmol) and ED (1.37 g, 22.74 mmol) for R1 and a solution of MV (13.21 g, 60.00 mmol) and Dom (5.34 g, 36.00 mmol) for R2 were added to a 250 mL round-bottomed flask and dissolved in 150 mL of DCM. The reactions were carried out in atmospheric conditions for 5 h. The resulting mixtures were washed and dried according to the procedure used for the methacrylation reactions.

### DLP 3D Printing of the Resins and Post-Treatment Strategies

After their synthesis, R1 and R2 were dissolved in DCM with a concentration of 70% w/v, and BAPO with a concentration of 5% w/w was added as a photoinitiator. The resulting solutions were subjected to DLP with a 3D printer (Asiga MAX X27 UV) endowed with a 385 nm light source. Build files and printing parameters were processed using Asiga Composer software. Based on the resulting working curves, R1 and R2 were printed using exposure times for each layer of 133 s (burn-in exposure time 151 s) and 67 s (burn-in exposure time 86 s), respectively; the layer thickness and the light intensity were set to 0.05 mm and 28.8 mW/cm^2^, respectively. Residual uncured resin was removed from the surfaces of the prints by washing them in two distinct DCM baths for 2 min. Finally, the prints were dried in a vacuum oven at 30 °C for 48 h. The so obtained thermosets photopolymerized from R1 and R2 were labeled T1 and T2, respectively.

After their production, the thermosets were post-treated to facilitate coating with lignosulfonate. Two distinct strategies were employed for the post-treatment. The first approach consisted of subjecting T1 and T2 to a 385 nm UV lamp in a postcuring chamber (Asiga Flash) for 6 min (3 min for each side). For the second strategy, both T1 and T2 were subjected to an atmospheric pressure non-equilibrium plasma treatment performed by using a dielectric barrier discharge (DBD) plasma source operating in environmental air and driven by a high-voltage micropulsed generator provided by AlmaPlasma srl. The DBD plasma source consisted of two parallel metal plates having a surface of 14 × 10 cm^2^. The upper electrode, covered with a ceramic layer as a dielectric, was connected to the high-voltage generator; while the lower electrode was grounded. The treatment was performed by placing the samples onto the grounded electrode and directly subjecting them to plasma discharge. The interelectrode gap was 1 mm, and the peak voltage and the pulse repetition rate were set to 8 kV and 5 kHz, respectively. Samples were exposed to plasma for 20 s (10 s for each side).

T1 and T2 subjected to the UV post-treatment were labeled T1_UV and T2_UV, respectively; while the samples subjected to the plasma post-treatment were labeled T1_Plasma and T2_Plasma, respectively.

### Preparation of Lignosulfonate-Coated Thermosets

T1 and T2, as well as all the post-treated thermosets, were subjected to a procedure aiming at modifying the materials with a lignosulfonate coating. The materials were immersed in an aqueous solution containing sodium lignosulfonate with a concentration of 3% w/v. Each sample was immersed in the solution for 2 min. Afterward, the sample was removed, immersed in distilled water for 1 min, gently wrapped in tissue paper to remove the excess of water, dried overnight at 60 °C, and then stored in a vacuum oven at 30 °C for 48 h before being characterized. The thermosets after the coating procedure were labeled T1_C, T1_UV_C, T1_Plasma_C and T2_C, T2_UV_C, and T2_Plasma_C, respectively.

### Characterizations

To confirm the chemical structure of the resins, ^1^H NMR spectra of R1 and R2 were performed on an Avance 400 (Bruker, U.S.A.) spectrometer (400 MHz). DMSO-*d*_6_ was used as the solvent and was also utilized as the internal standard for calibrating the chemical shift.

The chemical structures of R1 and R2 and of all the thermosets without and with coating were analyzed via a PerkinElmer Spectrum 2000 Fourier transform infrared (FTIR) spectrometer (Norwalk, CT) equipped with an attenuated total reflectance (ATR) sampling accessory. All the spectra were recorded in the wavenumber range of 4000–600 cm^–1^ using 16 scans at a resolution of 4 cm^–1^.

The solvent-resistant properties of all the printed thermosets were investigated against 4 common solvents (DCM, Acetone, DMF, and aqueous HCl 37% w/w) by immersing each thermoset (around 20 mg) in 2 mL of solvent for 72 h. Afterward, the sample was removed and dried in a vacuum oven at 60 °C until a constant weight was reached. The gel fraction for each sample was determined according to eq 1.
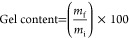
1where *m*_f_ is the final weight of the sample after 72 h of immersion and drying; while *m*_i_ is the initial weight of each sample before the immersion. The analysis was performed in triplicate for each sample and for each solvent.

The water contact angle of the printed thermosets was evaluated by using a contact angle analyzer Theta Lite (Biolin Scientific), according to the sessile drop technique. All measurements were performed at room temperature using deionized water. A water droplet of 4 μL was placed on the surface of the thermoset, and the diameter of the droplet was collected after 10 s of the application via a video camera. The contact angle values were obtained using Laplace Young curve fitting based on the image of the water drop. The value of the statistical contact angle is an average of five values.

Tensile tests were performed on 3D-printed bars (26.00 mm × 1.53 mm × 0.75 mm) using an Instron 5944 instrument equipped with a 500 N load cell. Thickness for each analyzed sample was considered as the mean value of 5 different measurements performed along the specimen’s length. Samples were conditioned in a controlled environment with a temperature of 22 °C and 50% relative humidity for 2 days before testing.

Thermogravimetry analysis (TGA) of the printed thermosets was performed by a TGA/SDTA851e instrument (METTLER TOLEDO, U.S.A.). Samples with a weight of around 10 mg were inserted in 70 μL ceramic crucibles and subjected to a heating scan from 30 to 600 °C with a heating rate of 10 °C/min and under a 50 mL/min nitrogen flow.

Dynamic mechanical analysis (DMA) of the printed thermosets was performed by using a TA Instruments Q800 in tension film mode. The experiments, in multifrequency strain mode, were performed with a force track of 125% at a frequency of 1.0 Hz. The deflection amplitude of oscillation and Poisson’s ratio were set at 15 μm and 0.44, respectively. The samples were equilibrated at 25 °C for 2 min and tested from 25 to 170 °C at 1 Hz with an amplitude oscillation of 15 μm and at a 3 °C/min heating rate. The glass transition temperature (*T*_g_) was considered as the maximum of the tan δ curve. Each measurement was performed in triplicate.

The morphology of all of the thermosets without and with coating was investigated by means of an SU8220 SEM (Hitachi, Japan) equipped with an EDS detector (X-Max^N^, Oxford Instruments, UK), operating at an accelerated voltage of 10 kV. All of the thermosets were sputter-coated with 3.5 nm gold before the analysis.

The UV shielding properties and antioxidant activity of all the printed as well as coated thermosets were investigated by means of UV–vis spectroscopy (Shimadzu UV-2550 spectrophotometer). For the assessment of the UV shielding properties, the water solubility of lignosulfonate was exploited. Each sample was immersed in distilled water with a concentration of 2.8 g/L and kept in immersion for 72 h. After this period of time, the sample was removed, and the UV absorbance of the solution, where the sample was kept in immersion, was measured in a wavelength range of 280–500 nm.^38^

The antioxidant activity was investigated by using the DPPH° free radical scavenging method. Samples with a surface of 1 cm^2^ were immersed in 3 mL DPPH° in methanol (2 mg/L). A control sample consisting of only DPPH° in methanol was also prepared. The incubation was carried out at room temperature in the dark. After 10 min, 30 min, 1 h, and 2 h from the immersion, 300 μL were withdrawn from each solution, immediately replaced with methanol, and subjected to the measurement of the absorbance at 517 nm using a Shimadzu UV-2550 spectrophotometer (Shimadzu Corp., Kyoto, Japan). The percentage of residual DPPH° was calculated according to eq 2.^38,39^
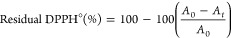
2where *A*_0_ and *A*_*t*_ are the absorbances of the control (methanolic DPPH°) and of the coated sample at the considered time, respectively. The experiment was performed in triplicate.

## Results and Discussion

**Figure 1:**
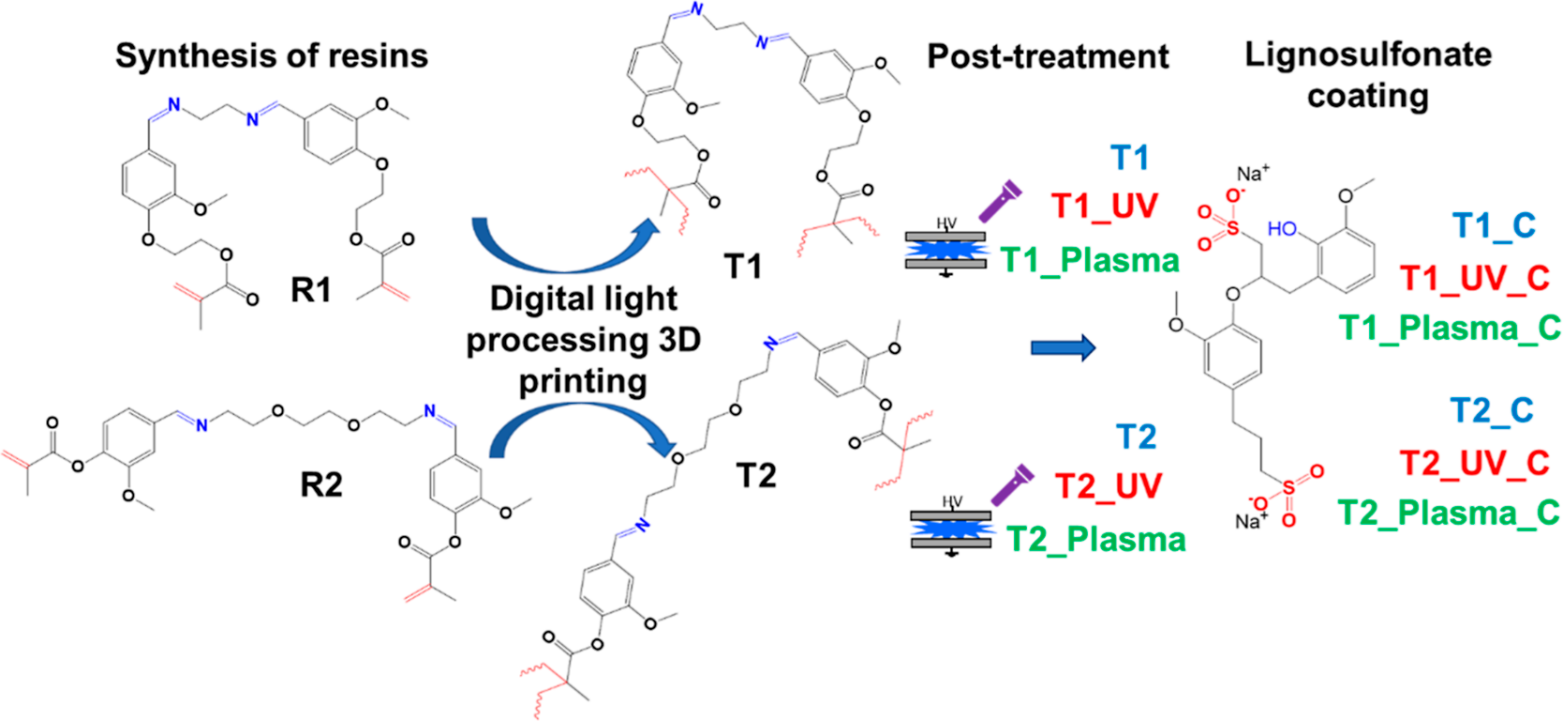
Scheme of the process summarizing the steps for the obtainment of the thermosets from the two resins, their post-treatment, and coating with lignosulfonate.

Two different post-treatment strategies, i.e., atmospheric pressure non-equilibrium plasma treatment and, as a comparison, the widely employed UV exposure, were evaluated to facilitate the coating of vanillin Schiff-base thermosets with a functional polymer coating. The scheme of the process is summarized in Figure 1. Before the coating procedure, the chemical, thermal, and mechanical properties of the post-treated thermosets were carefully investigated and compared with those of the as-printed samples.

### DLP 3D Printing of R1 and R2

Both R1 and R2 have as building blocks vanillin and a diamine, ED or Dom, respectively. The employment of short diamine, such as ED, in the vanillin Schiff-base resin requires the replacement of MV with M-ExtV for the obtainment of a coherent thermoset during the UV exposure, as demonstrated in our previous work.^9^ Conversely, the imination of MV with longer and more flexible diamine, Dom, enables the obtainment of a good quality nonfragile thermoset during the DLP photopolymerization. This can be explained by the higher molecular weight of this diamine with respect to ED (148 g/mol vs 60 g/mol), providing some flexibility in the material. The chemical structure of both resins was confirmed by means of ^1^H NMR (Figures S1 and S2) and ATR–FTIR (Figure S3).

The influence of the structure of the resins on the DLP printability was assessed by exploiting the Jacobs working curve model, which enables correlation of the penetration depth of the photons through the resin with the light irradiation dose required to induce photopolymerization (Figure S4). Additional details for the determination of the working curves are reported in the Supporting Information. The lower penetration depths of R1 with respect to R2 are beneficial for high print quality as they prevent overcuring caused by the diffusion of the incident light across undesired layers.

**Figure 2:**
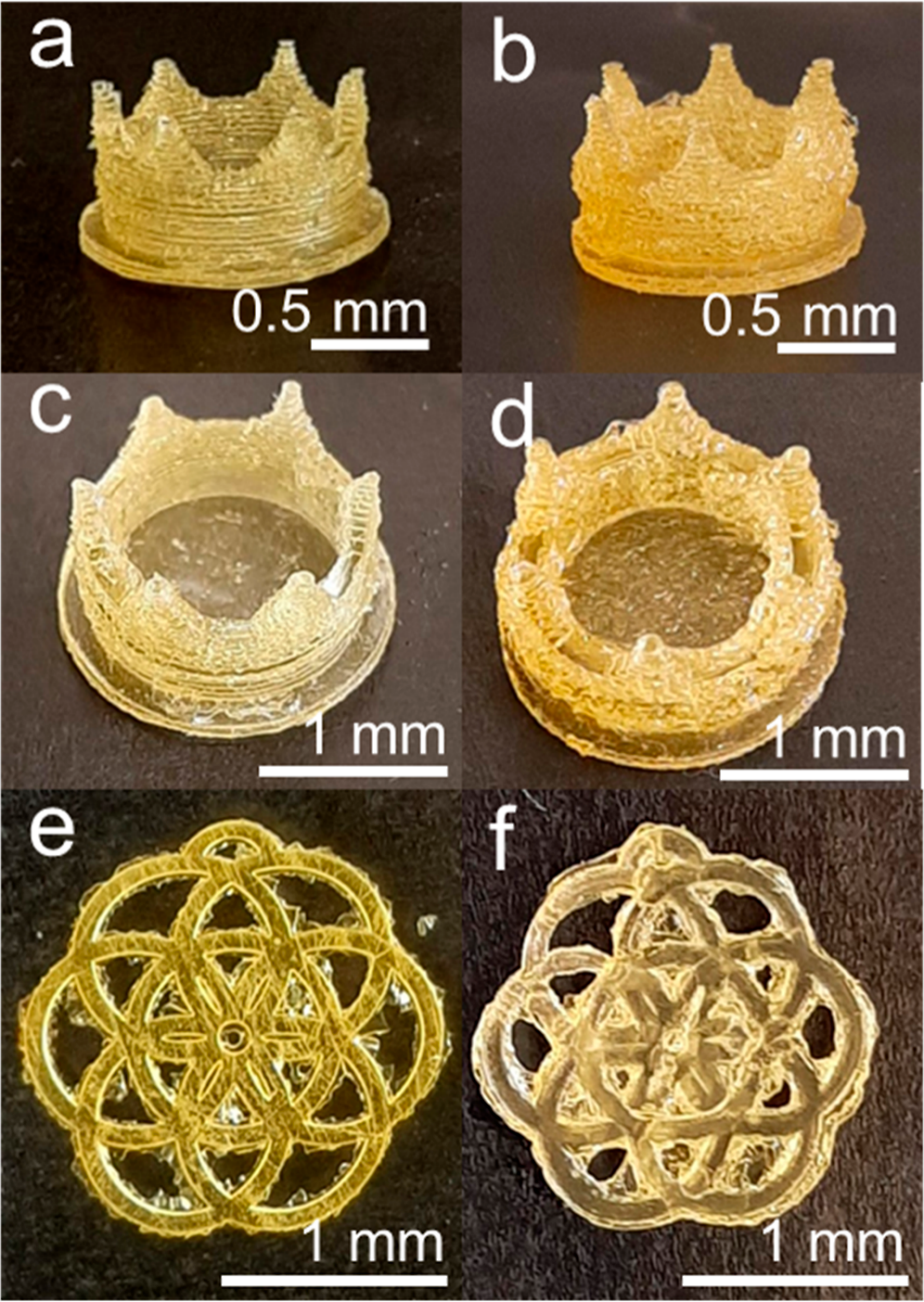
DLP 3D-printed crowns (a–d) and flowers (e,f) obtained from R1 (a,c,e) and R2 (b,d,f).

This was also practically confirmed from the observation of the characteristics of the DLP prints, i.e., a crown (Figure 2a–d) and a flower (Figure 2e,f). Indeed, the prints obtained from R1 (Figure 2a,c,e) show a higher resolution than those obtained from R2 (Figure 2b,d,f), which are clearly affected by the overcuring phenomena, observable in particular as the poorer definition of the crown perimeter and by the presence of undesired cured resin filling the parts of the flower supposed to be empty.

### Influence of the Different Post-Treatment Strategies

**Figure 3:**
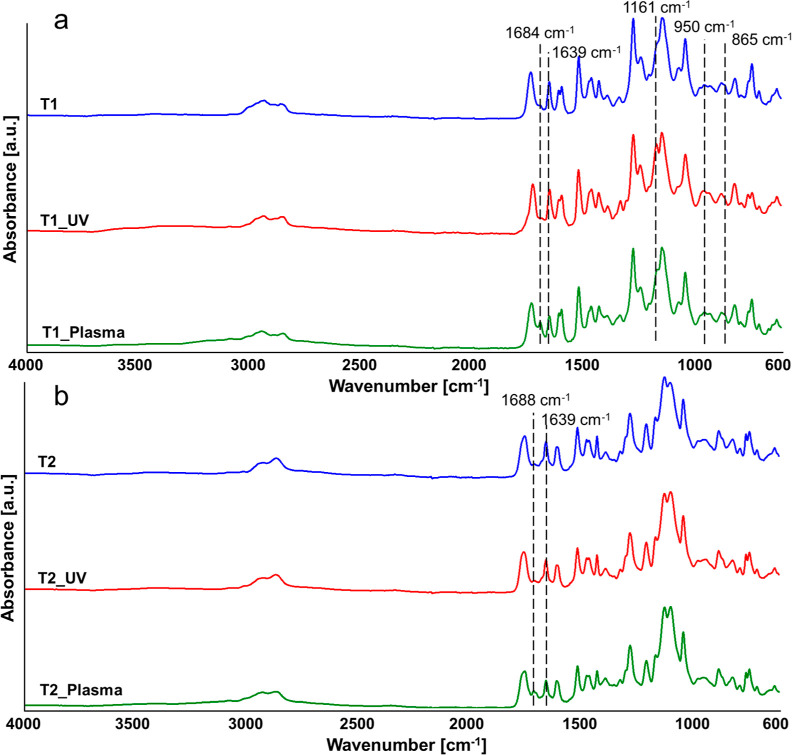
ATR–FTIR spectra of (a) T1, T1_UV, and T1_Plasma; (b) T2, T2_UV, and T2_Plasma.

The chemical structures of T1, T1_UV, and T1_Plasma, as well as those of T2, T2_UV, and T2_Plasma, were elucidated by means of ATR–FTIR. As observable in Figure 3, the DLP 3D printing and curing of R1 and R2 did not affect the Schiff-base linkages, since both T1 and T2 show the typical peak of the imine bonds at 1639 cm^–1^, while the aldehyde characteristic peak (at 1684–1688 cm^–1^), typical of MV and MExt-V resins, was undetectable in both thermosets. The post-treatment by UV exposure of T1 modified the absorption band at 950 cm^–1^, caused a reduction in the intensity of the band at 865 cm^–1^ with respect to the adjacent peak at lower wavenumbers, and led to an increase in the intensity of the peak at 1161 cm^–1^. These results suggest the reduction of remaining –C=C– bonds in the thermoset and, therefore, a completion of the curing process. Moreover, the higher intensity of the asymmetric –C–O– stretching vibration of the carbonyl groups (at 1161 cm^–1^)^40–42^ advocates for the occurrence of surface oxidation during the UV post-treatment of T1. Conversely, no such changes were observed in the spectra of T2_UV after the UV postcuring of T2.

Plasma treatment did not cause any changes in the –C=C– absorption band or modifications of the –C–O– absorption peak for the two thermosets with respect to the FTIR spectra of the original samples. On the other hand, the appearance of the peak at 1684–1688 cm^–1^ and a decrease of the intensity of the imine peak at 1639 cm^–1^ with respect to the adjacent signal at lower wavenumbers clearly indicate the opening of some imine linkages with the consequence reformation of the aldehyde functions in both T1_Plasma and T2_Plasma.

These results highlight that the UV post-treatment induces significant modifications in the chemical structure of T1, which reaches a more complete curing during this step. The absence of similar changes in the FTIR spectrum of T2 after UV post-treatment might be explained by the complete curing of the resin already during the printing step. The plasma treatment did not have any noticeable effect on the –C=C–. It mainly caused the opening of some Schiff-base linkages in both thermosets.

**Figure 4:**
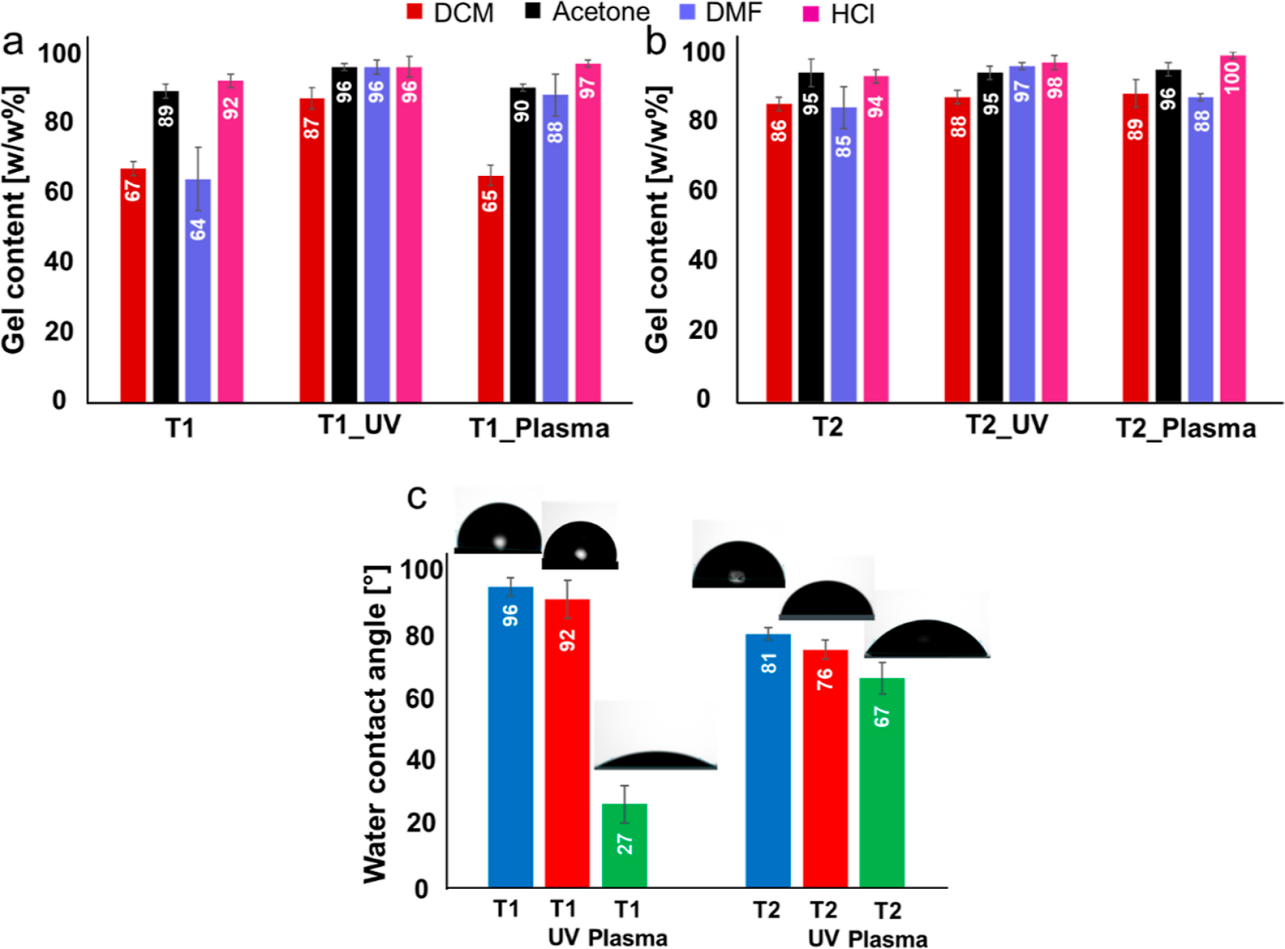
Gel content of (a) T1, T1_UV, and T1_Plasma; (b) T2, T2_UV, and T2_Plasma. (c) Water contact angle of all of the thermosets.

The gel content measurements (Figure 4) supported the interpretation of the ATR–FTIR results. After the UV post-treatment of the T1 thermoset (Figure 4a), a general improvement of the solvent resistance properties of T1_UV, particularly when the test was performed in DCM and DMF, was registered. On the other hand, no relevant differences were observed between T1 and T1_Plasma, except for an increase of the solvent resistance to DMF. In agreement with the FTIR analysis, the exposure of T1 to the UV completes the curing and increases the cross-linking density of the thermoset, with a consequent improvement of the solvent-resistant properties. The plasma treatment did not significantly contribute to the curing of the thermoset, and the induced opening of some imine bonds turned out not to affect the solvent-resistant properties of the thermoset.

In the case of the T2 thermosets (Figure 4b), all the thermosets showed similar solvent-resistant properties, confirming that, differently from T1, T2 had already reached a higher degree of curing during the printing process, and no additional improvements in this sense are imparted by the post-treatment process. In light of these results, the post-treatment of T2 with the aim to complete the curing is not necessary.

The water contact angle measurements (Figure 4c) documented the absence of important variations in the thermosets’ surface hydrophilicity before and after UV post-treatment. This result can be explained by considering that no significant structural modifications were observed on T2 and that no changes in the polar functional group were introduced on the surface of T1 during the completion of the curing. Conversely, both plasma postcured thermosets show a significant decrease of the water contact angle with respect to the original samples. This phenomenon is ascribed to the presence in the plasma discharge of atomic and molecular diatomic charged particles, electrons, and neutrals derived from the ionization of gases, able to interact with the thermosets.^43,44^ In particular, the energetic plasma species are able to cleave the covalent bonds of the surface layer, leading to the formation of radicals and/or to the generation of new functional groups.^45^ In our case, this effect is observed in the reformation of aldehyde and amine groups, which increases the hydrophilicity of the surfaces. The greater water contact angle reduction observed for T1_Plasma is supported by ATR–FTIR spectra showing greater intensity for the aldehyde band, indicating that a larger amount of imine bonds was opened in T1_Plasma compared to T2_Plasma (Figure 3).

**Figure 5:**
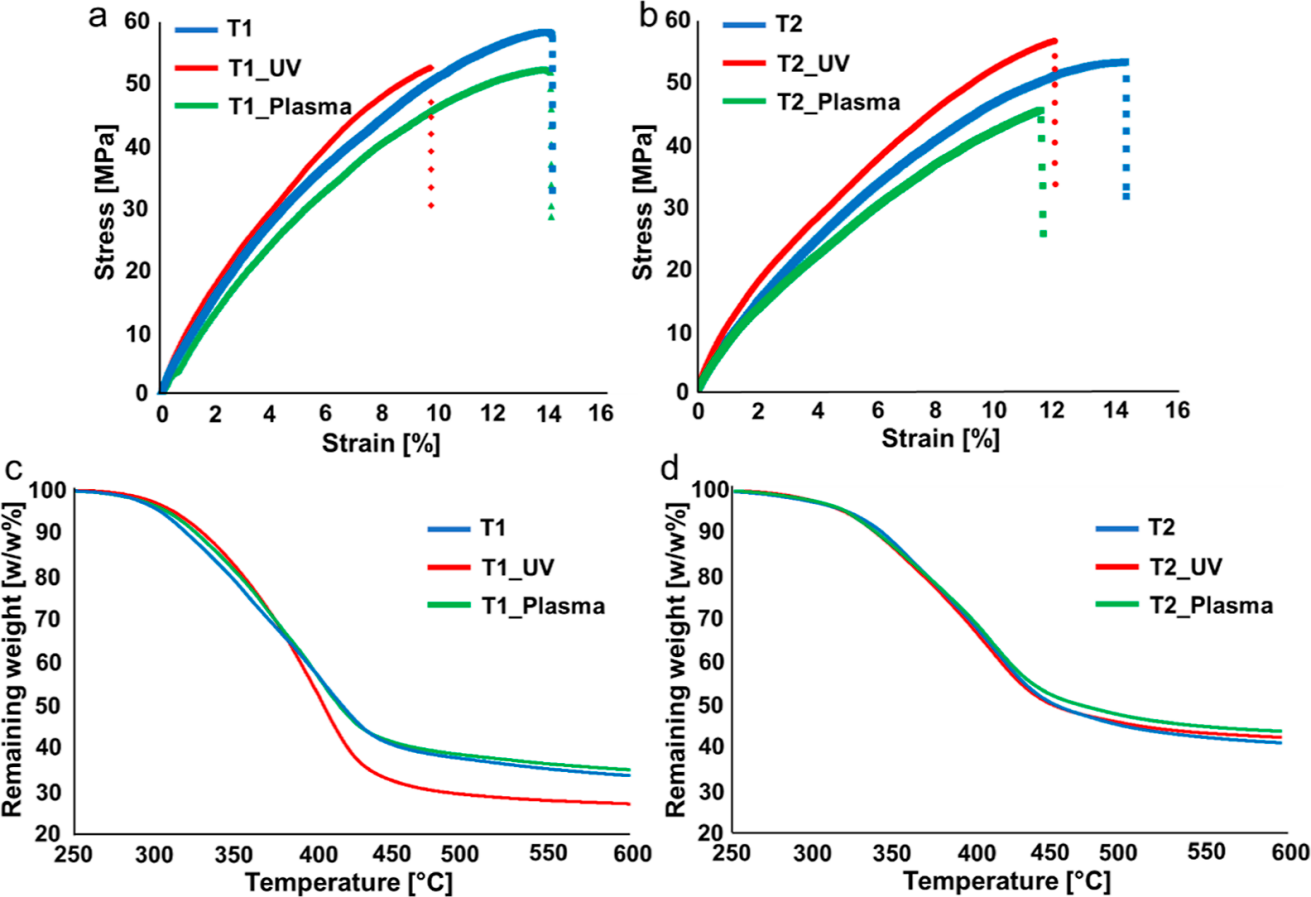
Stress–strain measurements performed on (a) T1, T1_UV, and T1_Plasma and (b) T2, T2_UV, and T2_Plasma. TGA analysis was carried out on (c) T1, T1_UV, and T1_Plasma and (d) T2, T2_UV, and T2_Plasma.

Besides the surface hydrophilicity, the opening of the imine bonds induced by the plasma post-treatment turns out to affect the mechanical properties of the thermosets (Figure 5a,b, and Table S1). Indeed, as a result of the opening of the imine bonds, a decrease of the elastic modulus is registered for both T1_Plasma and T2_Plasma with respect to T1 and T2, respectively; additionally, a reduction of the stress at break is observed for the T2_Plasma. The UV post-treatment did not significantly affect the resulting mechanical properties, except for a slight increase of the elastic modulus for both thermosets and a slight reduction of the stress at break for T1, in tune with the completion of the curing process. The comparison of the mechanical properties of DLP 3D-printed T1 and T2 with the thermosets obtained by UV lamp curing of corresponding resins, R1^9^ and R2,^11^ respectively, revealed the effectiveness of the DLP in terms of the degree of curing of the thermoset. Indeed, elastic moduli around 1 GPa were obtained for printed T1 and T2; which is significantly higher than the values registered for thermosets cured from corresponding R1 and R2 resins by simple UV lamp curing (around 370 and 640 MPa, respectively). This is ascribed to the layer-by-layer photopolymerization approach of DLP, which enables the obtainment of thermosets uniformly cured along their three dimensions. Conversely, when the curing of the resin is performed with the UV lamp, the UV light is not able to penetrate through the whole thickness of the film, leading to thermosets characterized by nonuniform properties along their thickness.

The TGA analysis (Figure 5c,d, and Table S2) did not highlight any significant differences in terms of *T*_deg 5%_ and *T*_deg max_ among the considered thermosets. On the other hand, some considerations can be made from the analysis of the residual weights at 600 °C. As shown, the T2, T2_UV, and T2_Plasma present similar residue at the end of the measurement; conversely, some differences can be noted among the different T1 thermosets. Although less cross-linked than T1_UV, both T1 and T1_Plasma showed a slightly higher residue, which might find an explanation in the occurrence of an exchange reaction between the imine functions and the unreacted vinylic –C=C– available in these two thermosets during the analysis.^46–48^ This exchange might lead to the preservation of the total number of double bond functions in the network and, therefore, to a higher char residue with respect to T1_UV. Moreover, the higher content of oxidized functions in T1_UV could contribute to the observed lower residue at 600 °C. Indeed, the relatively lower thermal stability of these functions might favor the scission of the network, inducing also secondary reactions that can cause thermal decomposition.^49^

From the analysis of the storage modulus as a function of the temperature (Figure S5a,b), no significant differences were detected when comparing the as-printed thermosets with those subjected to the post-treatments. An increase of the *T*_g_ was instead registered for both T2_UV and T2_Plasma with respect to T2 (Figure S5 d and Table S2), suggesting that some modification of the network, not detectable from the ATR–FTIR, might take place during the postcuring. Interestingly, the *T*_g_ (86 ± 9 °C) value of T1 turned out to be similar to the *T*_g_ obtained previously for the thermoset derived by simple UV-lamp curing of the corresponding R1 resin (83 ± 2 °C),^9^ although there was a dramatic increase of the elastic modulus as discussed above. On the other hand, T2 exhibited a higher *T*_g_ (95 ± 3 °C) than the UV-lamp-cured thermoset obtained from the corresponding R2 resin (75 ± 1 °C),^11^ which agrees with the measured mechanical properties. The result suggests that the higher mobility of R2 resin with respect to R1 resin, due to the longer flexible diamine employed, also reflected higher chain mobility in the not completely cured networks, leading to the occurrence of the transition from the glassy state to the rubbery plateau at a lower temperature.

### Coating with Lignosulfonate

**Figure 6:**
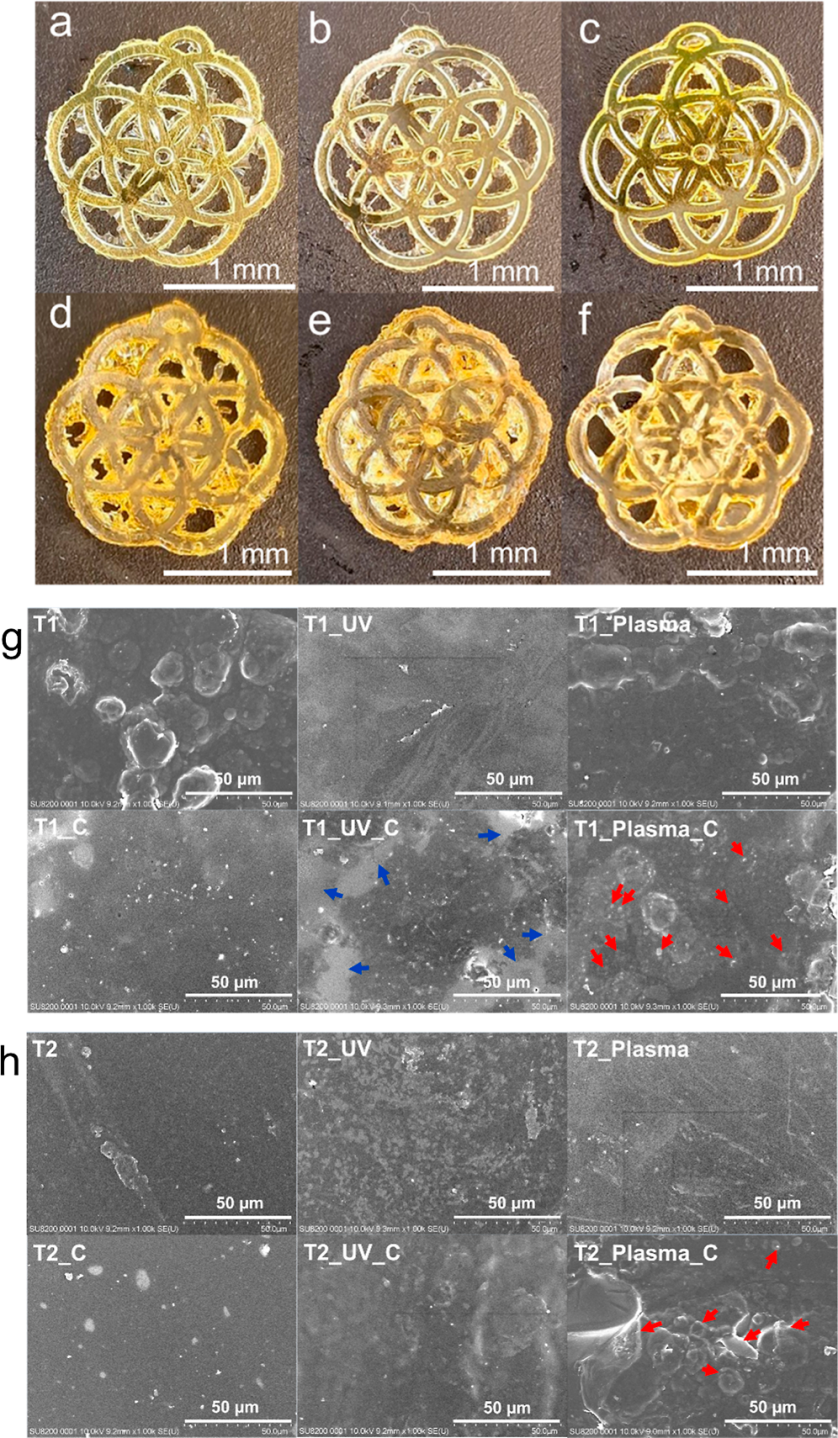
Printed flowers after the coating procedure: (a) T1_C; (b) T1_UV_C; (c) T1_Plasma_C; (d) T2_C; (e) T2_UV_C; (f) T2_Plasma_C. SEM images of (g) all the T1 thermosets without (upper line) and with (lower line) coating; (h) all the T2 thermosets without (upper line) and with (lower line) coating.

The effect of the post-treatment strategy on the attachment of the lignosulfonate coating on the thermoset surfaces was investigated. All the thermosets were subjected to the coating procedure described in the Experimental Section. As shown in Figure 6a–f, the appearance of the prints does not noticeably change after the lignosulfonate coating process; except a shift of color from a pale yellow (Figure 2f) to an intense yellow (Figure 6d–f) detectable for the T2 thermosets. Moreover, the coating does not fill the empty areas of the prints, as no relevant variations in the aspect of the prints can be observed from the comparison of Figure 6 with Figure 2e,f. The presence of undesired material in the parts of the print that should have been empty (see in particular Figure 6d–f) can be ascribed to the overcuring of R2 during the DLP, as described above.

The SEM analysis of the thermoset morphology documented the role of the post-treatment strategy for the effective coating of the surface with lignosulfonate. As observable in Figure 6g,h, T1 and T2 show bubble-like and smooth morphologies, respectively, which are not altered by the plasma treatment. The UV post-treatment results in the disappearance of the bubble-like morphology of T1 in favor of a smooth and homogeneous surface, while a significant roughness increase is observed for T2. The transition from bubble-like to smooth surface morphology might agree with the completion of the curing during the UV post-treatment of unreacted monomers present on the surface of T1. The modification of morphology induced in T2 by the UV post-treatment could be explained by referring to the capacity of the UV light to generate, when operated in the presence of oxygen, singlet molecular oxygen ^1^O_2_ able to interact with the polymeric surface, inducing an increase of the roughness.^50^

A lignosulfonate coating was successfully attached on the surface of the two plasma post-treated samples. This is documented by the presence of uniformly distributed micrometric round-shaped features (as those highlighted by the red arrows in Figure 6g) on T1_Plasma_C and by the formation of tridimensional structures (red arrows in Figure 6 h), suggesting the presence of a thick coating on T2_Plasma_C. A coating can be detected also on T2_UV_C, as documented by its lower roughness with respect to T2_UV. Concerning T1_UV_C, the surface of this sample shows an inhomogeneous aspect characterized by coated areas with a morphology similar to T2_Plasma_C and uncoated areas (highlighted by blue arrows in Figure 6g), suggesting a lower and nonuniform adhesion of lignosulfonate on the surface of T1_UV with respect to T1_Plasma. While the T2 morphology did not vary after the coating procedure, the absence of the bubble-like morphology in T1_C is likely ascribed to the removal of the unreacted monomer still present on the T1 surface during the dip coating procedure. Finally, the formation of a higher lignosulfonate amount on the surface of the plasma post-treated samples is also confirmed by the significant increase of weight registered for these samples after the coating procedure, as documented in Table S3.

In light of these results, the presence of polar amine functional groups and aldehydes deriving from the opening of the Schiff-base linkages during the post-treatment turns out to be beneficial for the interfacial interactions between the 3D-printed thermosets and the coating and subsequently the formation of a compact and uniform coating on the thermoset surface, as further explained in the following.

**Figure 7:**
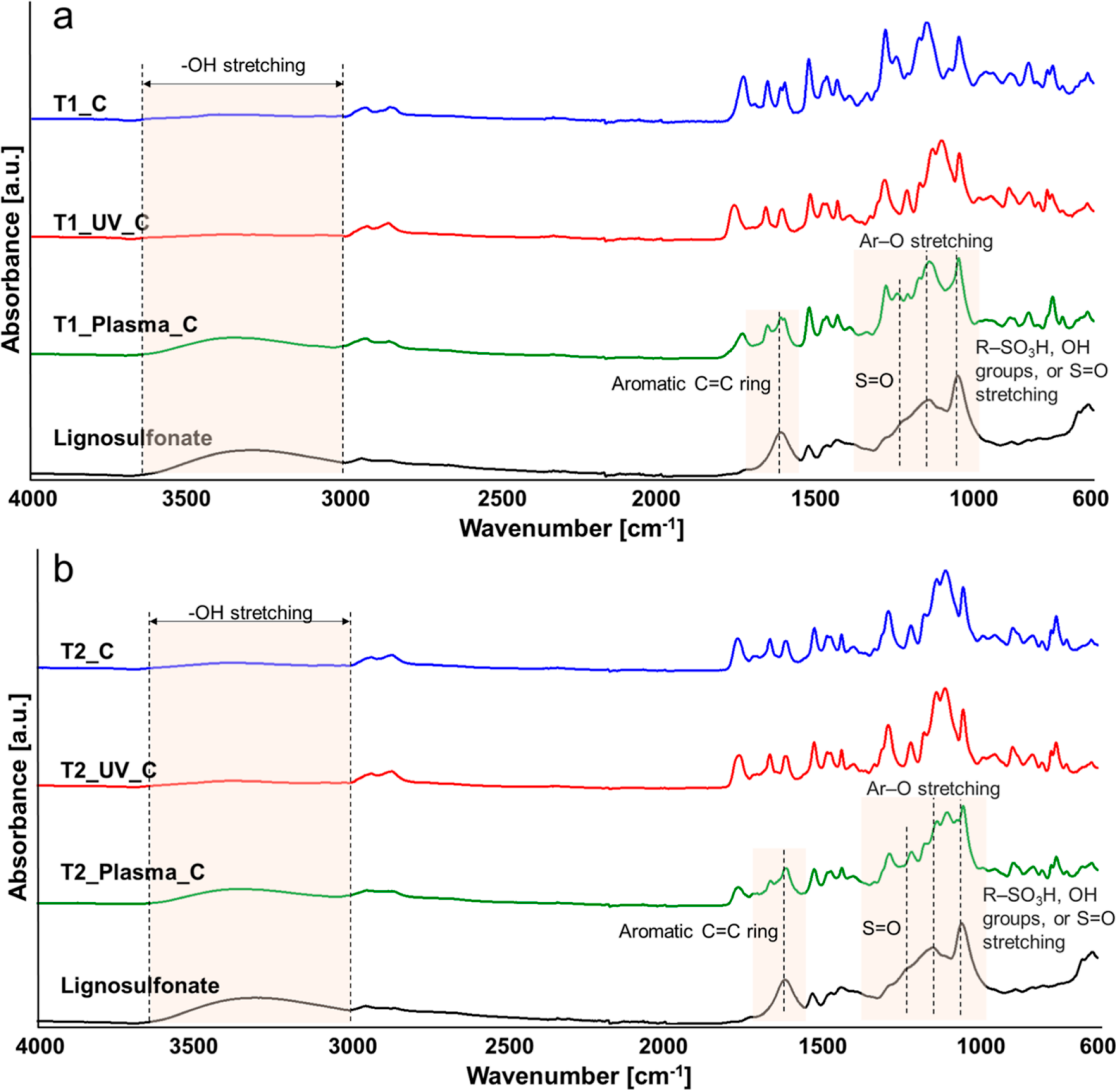
ATR–FTIR spectra of (a) T1, T1_UV, and T1_Plasma after the coating procedure; (b) T2, T2_UV, and T2_Plasma after the coating procedure; the characteristic peaks of lignosulfonate have been highlighted.^51^

The presence of a coating on the surface of the thermosets was further confirmed by means of ATR–FTIR (Figure 7) and EDS (Table 1). A modification of the ATR–FTIR spectra was registered for all of the thermosets subjected to the coating procedure; however, in agreement with the SEM results, these changes were more evident for the plasma post-treated thermosets. Indeed, both T1_Plasma_C and T2_Plasma_C showed a very intense band in the region between 3670 and 3000 cm^–1^ ascribed to –OH stretching vibration; a modification of the spectrum in the range 1640–1584 cm^–1^ and between 1300 and 974 cm^–1^ due to an overlapping of the signals of the thermoset with those of the lignosulfonate (Figure S6). An additional comparison among the FTIR spectra of the thermosets before and after the coating procedure with the spectrum of the lignosulfonate is reported in Figures S7–S12. Although some morphological changes were evident from the comparison of the SEM images of noncoated and coated UV post-treated thermosets; except for a slight increase of the intensity of the –OH band, no characteristic lignosulfonate signals were detected in the FTIR spectra, in tune with the predicted poor adhesion of the coating on the UV post-treated thermosets. Similar results were obtained for T1_C and T2_C, confirming that the change of morphology detected after the coating procedure of T1 cannot be ascribable to any significant adhesion of the coating.

**Table 1:** EDS Analysis of All the Thermosets without and with Coating Expressed in Atomic Percentage with Respect to the C Atomic Concentration

sample	O/C [at. %]	N/C [at. %]	S/C [at. %]
T1	34.68	9.67	0.03
T1_UV	31.58	8.58	0.06
T1_Plasma	39.60	9.48	0.03
T1_C	32.94	9.51	0.04
T1_UV_C	28.88	5.72	0.20
T1_Plasma_C	43.97	9.31	0.55
T2	39.03	9.25	0.00
T2_UV	32.74	10.05	0.00
T2_Plasma	35.00	10.56	0.09
T2_C	34.14	8.05	0.06
T2_UV_C	30.15	8.78	0.00
T2_Plasma_C	35.14	11.01	0.23

The EDS characterization (Table 1) supported the ATR–FTIR results, highlighting the presence of the highest contents of sulfur groups, a characteristic of lignosulfonate, on the surface of both T1_Plasma_C and T2_Plasma_C.

The more intense lignosulfonate signals detected by both FTIR and EDS analysis of the plasma post-treated thermosets can be associated with the presence of a greater amount of coating on the surface of these samples with respect to the original or UV post-treated thermosets. This result might be explained by considering that lignosulfonate is an anionic polymer characterized by a relevant hydrophobic aromatic component, some weakly ionized groups (such as carboxyl and phenolic hydroxyl groups), and polar sulfonic acid groups.^52,53^ These characteristics make this polymer able to give place to noncovalent interactions with other polymers. The presence of polar functionalities such as aldehyde and amine, both deriving from the opening of the imine bonds in T1_Plasma and T2_Plasma, enables the establishment of a larger number of secondary interactions between these thermosets and the coating compared with those generated by the T1, T2, and the respective UV postcured thermosets.

**Figure 8:**
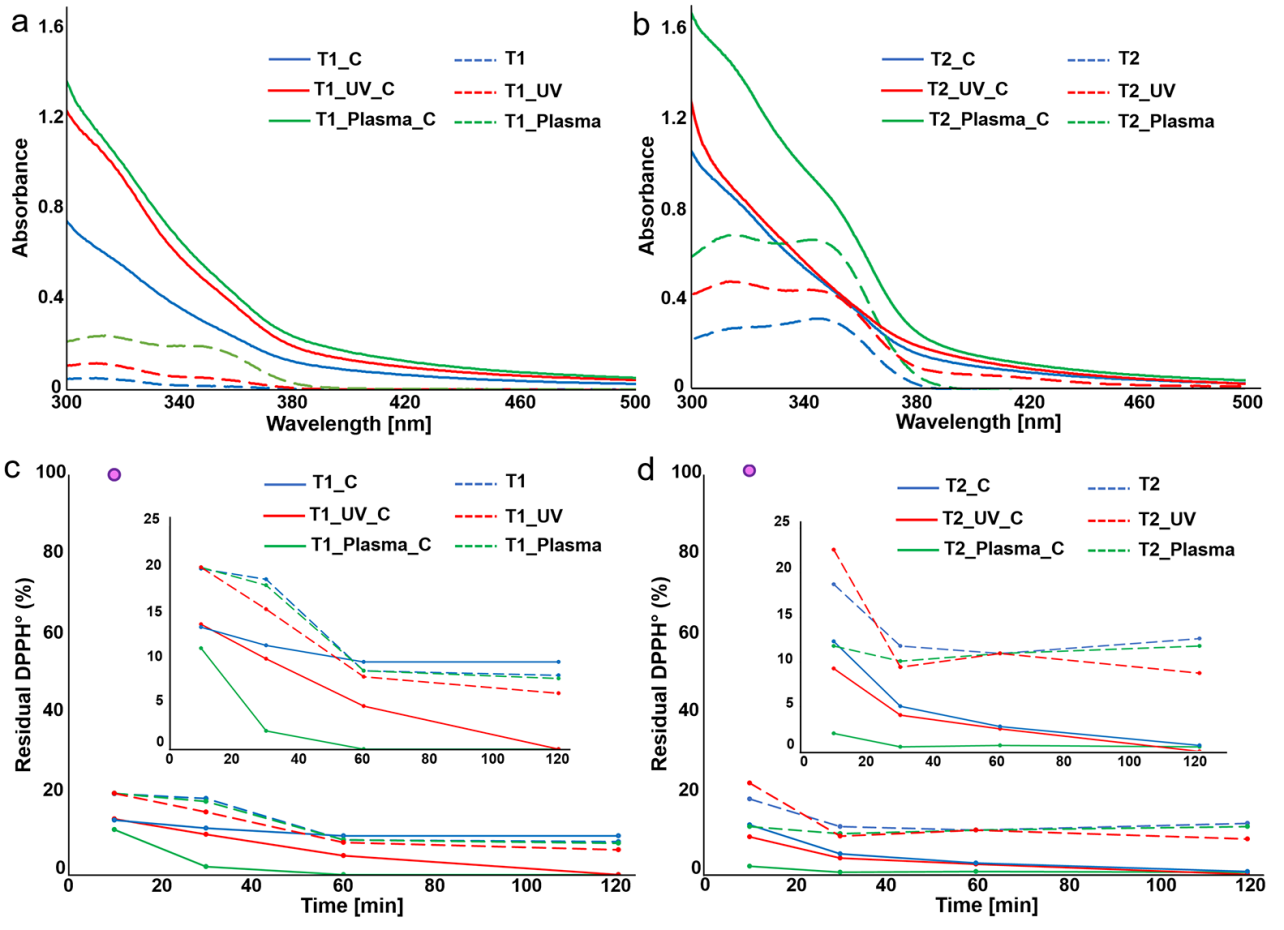
UV shielding properties of (a) T1, T1_UV, and T1_Plasma without and with coating; (b) T2, T2_UV, and T2_Plasma without and with coating. Antioxidant properties of (c) coated T1_C, T1_UV_C, and T1_Plasma_C and (d) coated T2_C, T2_UV_C, and T2_Plasma_C. The pink dot relates to the methanolic DPP*H*° after 10 min from the preparation of the solution.

As observable from Figure 8a,b, the aromatic structure of lignosulfonate renders this polymer an effective natural UV blocker. Evident UV-shielding properties were detected in particular for both plasma post-treated thermosets after the coating procedure, consistent with the presence of a thicker and more uniform layer of lignosulfonate on the surface of these samples. At the same time, it is interesting to notice that all T2 thermosets showed better UV-shielding properties than their T1 counterparts, similar to the results obtained for the noncoated samples. The higher UV blocking properties of T2 with respect to T1 can be linked to the presence of ether groups;^54^ while the reformed aldehyde functions might explain the better properties of T1_Plasma and T2_Plasma with respect to the other noncoated thermosets.

The ATR–FTIR analysis (Figures S13 and S14) performed on T1_Plasma_coating and T2_Plasma_coating after the 72 h of immersion in water before the assessment of the UV-shielding properties highlights some modification of the spectra with respect to those collected on nonimmersed samples. However, some of the characteristic signals ascribed to lignosulfonate are still present after the prolonged immersion in water. It is possible, therefore, to assume that the UV-shielding properties come from a partial solubilization of the coating during the 72 h immersion in water; however, after this time, the coating is still present on the surface of the plasma postcured thermosets, suggesting the possibility to employ these materials for long-term applications.

The antioxidant properties of the noncoated and coated thermosets were assessed via 2,2-diphenyl-1-picrylhydrazyl (DPPH°) assay (Figure 8c,d). Focusing on the coated thermosets, the plasma post-treated samples show the highest antioxidant activities, clearly evident after 30 min for T1 and after only 10 min for T2, in tune with the presence of a higher amount of coating on the surface. The analysis of the other coated thermosets highlighted that at 10 and 30 min, both T2_C and T2_UV_C have higher antioxidant activity compared with T1_C and T1_UV_C. Since no significant differences were observed among the noncoated samples (Tables S4 and S5), also this result might be linked to the quantity of lignosulfonate coating on the thermoset.

Interestingly, the results clearly show that all the noncoated thermosets present high antioxidant properties, with a residual DPPH° in the solution lower than 25–30% after only 10 min from the sample immersion; this result might be connected to their vanillin-derived structure and to the presence of Schiff-base linkages.^55^

## Conclusions

We present a facile and simple non-equilibrium atmospheric plasma post-treatment strategy for the surface coating of DLP 3D-printed structures. First, two biobased Schiff-base resins, R1 and R2, were successfully obtained by the imination of methacrylated extended vanillin with ethylene diamine and methacrylated vanillin with 2,2′-(ethylenedioxy)bis(ethylamine), respectively. Both resins could be 3D-printed DLP onto tridimensional thermosets labeled T1 and T2, respectively. T1 showed a higher printing fidelity than T2, as confirmed by the reduced overcuring caused by the diffusion of the incident light across undesired layers in T1. None of the applied post-treatment strategies, UV light and non-equilibrium plasma treatment, significantly affected the surface morphology of the thermosets, except for some increase of the surface roughness. After UV-treatment, the characterization of the chemical properties of the thermosets documented the completion of the curing, a slight surface oxidation, and the increase of the solvent-resistant properties for T1_UV with respect to T1, while no relevant alterations of these characteristics were observed in T2_UV. The result suggests the potential of the UV post-treatment in inducing a completion of the curing of T1, which was further supported by the stress–strain results. The absence of significant variation of properties after the UV post-treatment of T2 is attributed to the reaching of a high degree of curing already during the printing, probably due to the greater flexibility and mobility of the diamine employed for the synthesis of the resin.

Differently from the UV light post-treated thermosets, FTIR analysis indicated that the plasma post-treatment mainly affected the Schiff-base linkages of the thermosets, leading to an opening of some of these bonds with the consequent reformation of aldehydes and amines already after 10 s of exposure. This is further supported by the water contact angle measurements, which documented a noticeable increase in the surface hydrophilicity upon plasma exposure. No relevant variations of the solvent-resistant properties were observed by comparing the plasma post-treated thermosets with the original samples, while a slight decrease of the mechanical properties was registered after this post-treatment.

The plasma post-treatment facilitated the attachment of lignosulfonate coating on the surface of both thermosets, which was documented by FTIR and SEM–EDS analysis. Some coating was also detected on T1_UV_C and T2_UV_C, although with a lower amount and homogeneity with respect to the plasma post-treated samples. The higher lignosulfonate content observed on the surfaces of T1_Plasma_C and T2_Plasma_C can be linked to the regenerated aldehydes and amino groups, facilitating favorable secondary interactions with the lignosulfonate. The coating endowed the thermosets with high UV shielding and antioxidant properties, mainly ascribed to the presence of phenolic functions in the lignosulfonate structure. At the same time, the uncoated thermosets also exhibited some antioxidant properties, probably in tune with their aromatic structure and the presence of Schiff-base linkages in the network. This paves the way for the development of 3D structures with active surfaces.

## Supporting Information Available

^1^H NMR of R1 and R2 resins; ATR–FTIR of R1 and R2 resins; working curves for the printing of both the resins; mechanical and thermal properties of all the thermosets before and after post-treatments; DMA analysis of all the thermosets; weight increase of the thermosets after the lignosulfonate coating procedure; ATR−FTIR spectrum of lignosulfonate; comparison of ATR–FTIR spectra of T1, T1_C, and lignosulfonate; comparison of ATR–FTIR spectra of T1_UV, T1_UV_C, and lignosulfonate; comparison of ATR–FTIR spectra of T1_Plasma, T1_Plasma_C, and lignosulfonate; comparison of ATR–FTIR spectra of T2, T2_C, and lignosulfonate; comparison of ATR–FTIR spectra of T2_UV, T2_UV_C, and lignosulfonate; comparison of ATR–FTIR spectra of T2_Plasma, T2_Plasma_C, and lignosulfonate; ATR–FTIR spectra of T1_Plasma_coated immersed in water for 72 h, T1_Plasma_coated and T1_Plasma; ATR–FTIR spectra of T2_Plasma_coated immersed in water for 72 h, T2_Plasma_C and T2_Plasma; residual DPPH° expressed as an average value with standard deviation for all the T1 samples without and with coating; and residual DPPH° expressed as an average value with standard deviations for all the T2 samples without and with coating (PDF)
