# Supplementary material for: Simple Non-Equilibrium Atmospheric Plasma Post-Treatment Strategy for Surface Coating of Digital Light Processed 3D-Printed Vanillin-Based Schiff-Base Thermosets
Source: ACS Appl Polym Mater. 2023 Sep 25;5(10):8506–17. doi: 10.1021/acsapm.3c01632 (PMC10580284; doi:10.1021/acsapm.3c01632)
Supplement: Supplementary file 1 — ap3c01632_si_001.pdf [file ap3c01632_si_001.pdf]

Supporting Information

Simple Non-Equilibrium Atmospheric Plasma Post-Treatment Strategy for Surface Coating of Digital Light Processed 3D-Printed Vanillin-Based Schiff-Base Thermosets

Anna Liguori[a,*], Huan Xu[b], Doli Hazarika[a], Minna Hakkarainen[a]

[a] KTH Royal Institute of Technology, Department of Fibre and Polymer Technology, Teknikringen 58, 100 44 Stockholm, Sweden

[b] School of Materials Science and Physics, China University of Mining and Technology, Xuzhou 221116, China

*liguori@kth.se

This supporting information file contains 14 Figures and 5 Tables on 12 pages.

Supporting Information

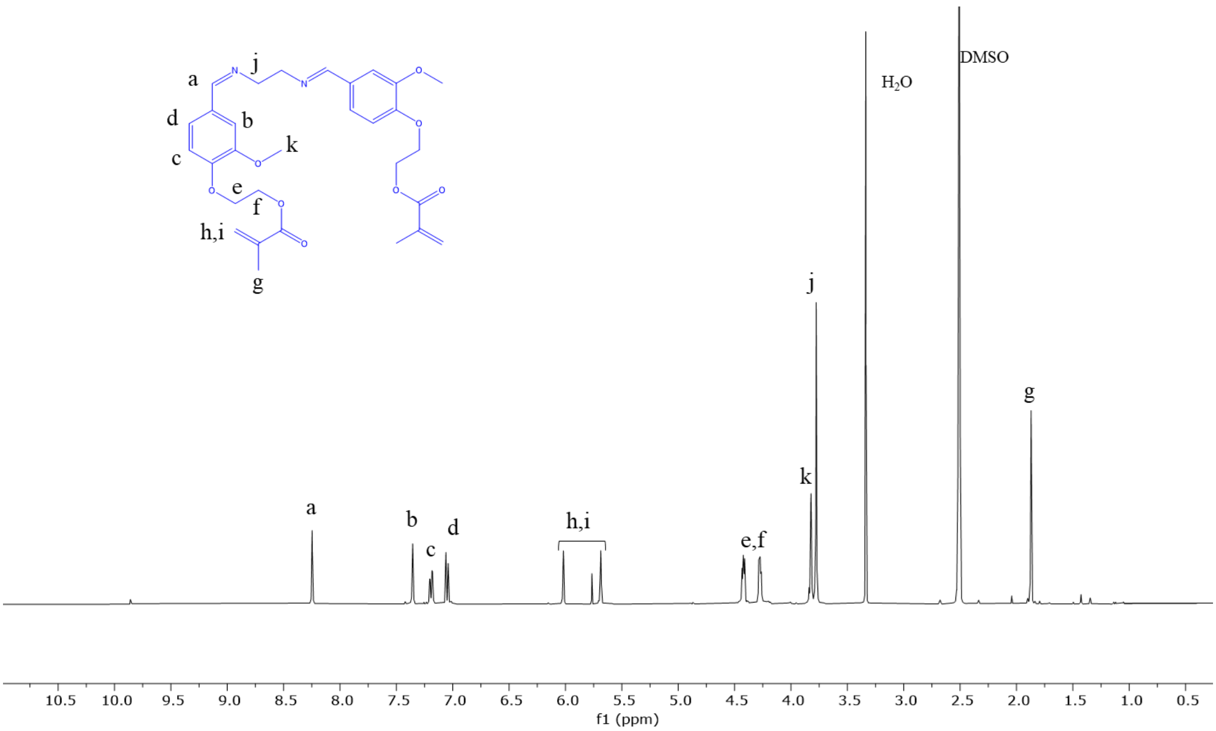

Fig S1. ^{1}H NMR spectrum of R1 resin.

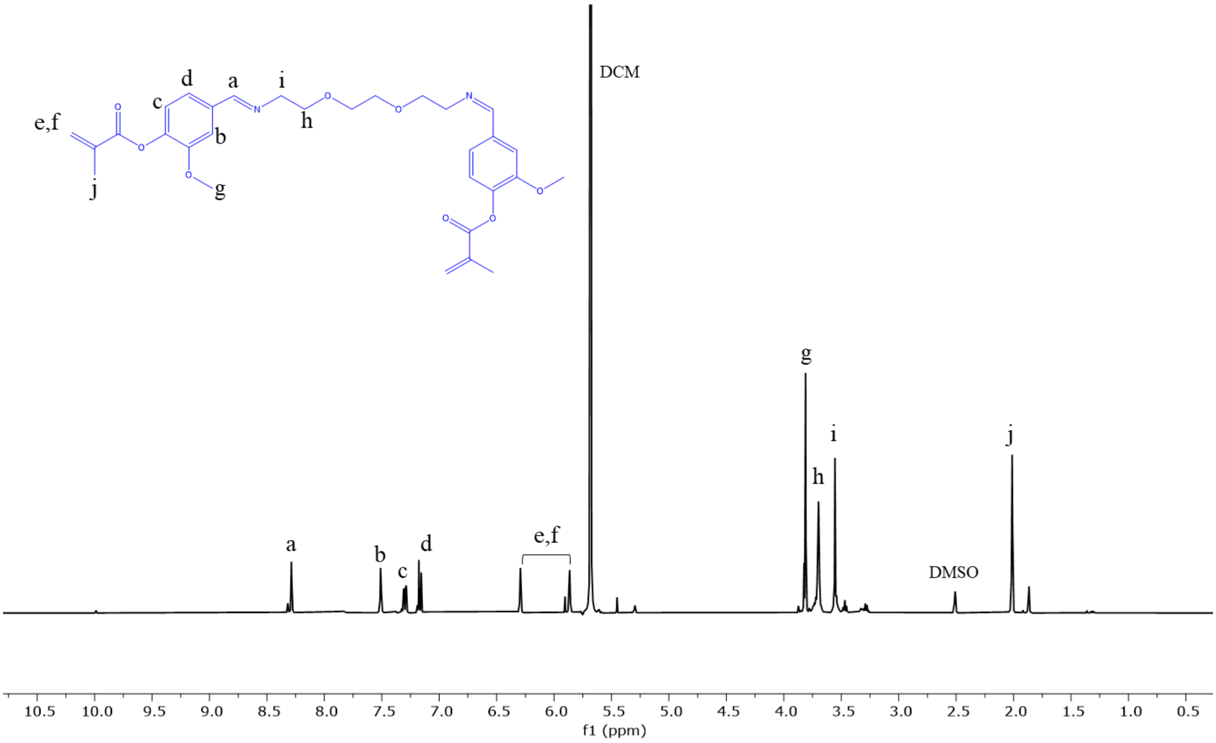

Fig S2. ^{1}H NMR spectrum of R2 resin.

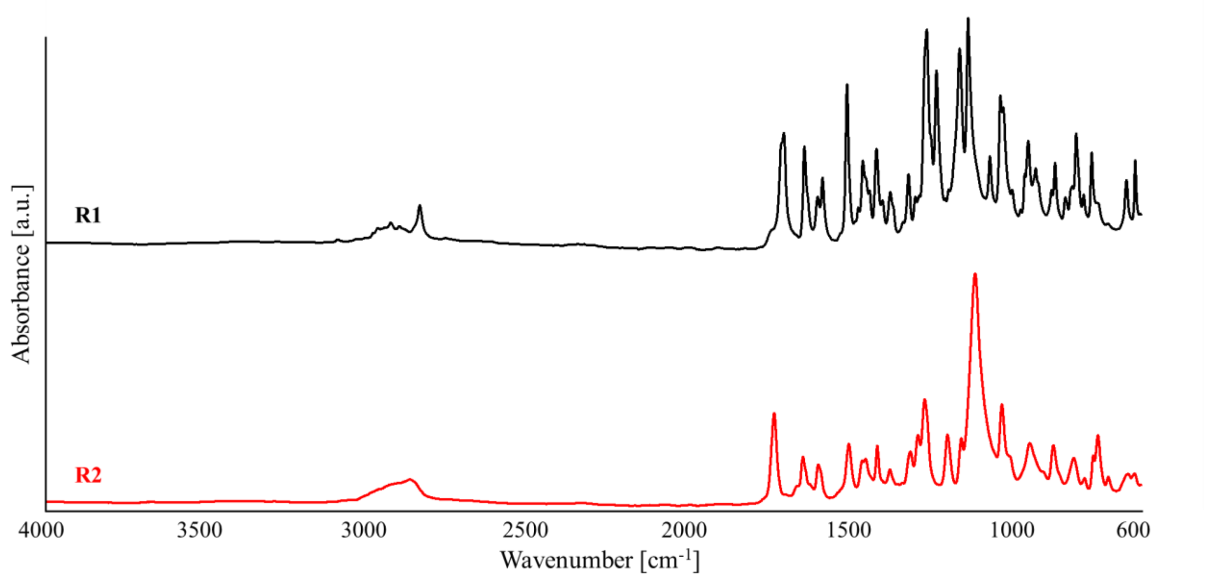

Figure S3. ATR-FTIR spectra of R1 and R2 resins.

Determination of the resins' working curves. The cure parameters of the resins were determined by implementing the Jacobs working curve model according with equation 1. The method correlates the resins' penetration depth (D_p) indicating how far light can penetrate into the resin, with the critical dose (E_{crit}), intended as the amount of light required for polymerization to proceed until a solid material is formed. Disks (3 mm in diameter) of each resin formulation were cured over the screen of the DLP 3D printer by exposing a small circle of light (385 nm, 28 mW/cm^2) at varying durations (from 80 s to 100 s). After a washing in DCM and a UV lamp post-curing (3 min/side), the thicknesses of the cured samples (C_d, µm) were measured using a micrometer thickness gauge. Thickness measurements were performed in triplicate for each sample. Each resin's working curve is obtained as a semi-logarithmic plot of C_d vs light irradiation dosage (E_{max}, mJ/cm^2), which is the product of the UV light intensity (mW/cm^2) and exposure time (s).

$$C_d = D_p ln \frac{E_{max}}{E_c} \quad \text{(equation 1).}$$

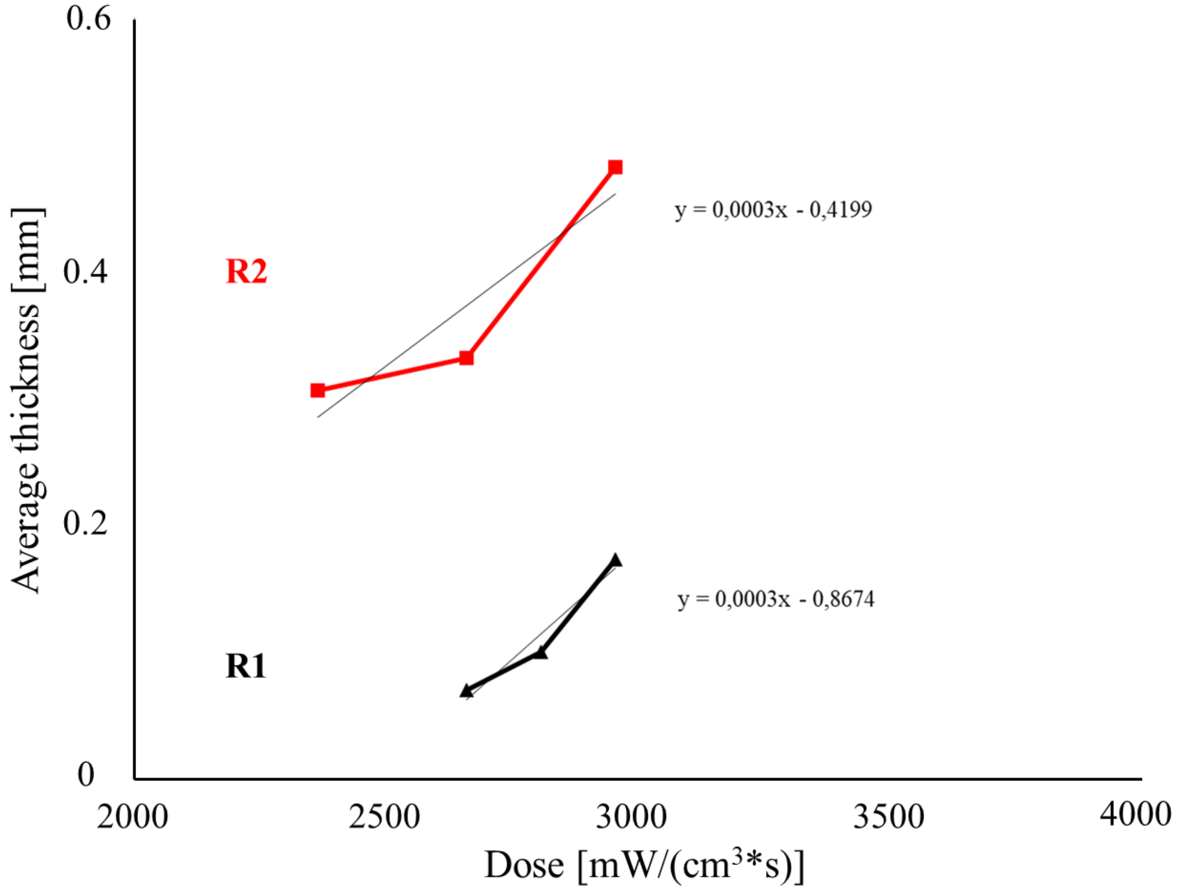

Figure S4. Working curves for the printing of R1 and R2 resins.

Table S1. Mechanical properties of all the thermosets before and after post-treatments.

**Sample**	**Strain at break [%]**	**Stress at break [MPa]**	**Elastic Modulus [MPa]**
T1	15±1	53±3	1207±166
T1_UV	10±3	51±7	1273±156
T1_Plasma	14±2	55±2	1064±146
T2	15±2	54±1	938±50
T2_UV	11±3	51±5	1190±91
T2_Plasma	12±1	43±4	911±48

Table S2. Thermal properties of all the thermosets before and after post-treatments.

**Sample**	T_g **[°C]**	$T_{deg5\%}$ **[°C]**	T_{degmax} **[°C]**	**Residue [w/w%]**
T1	86±9	306±4	414±18	34±6
T1_UV	87±4	308±3	414±5	26±2
T1_Plasma	87±8	308±3	426±4	35±6
T2	95±3	322±3	422±3	41±2
T2_UV	101±1	320±3	419±5	41±3
T2_Plasma	102±5	323±1	423±2	41±3

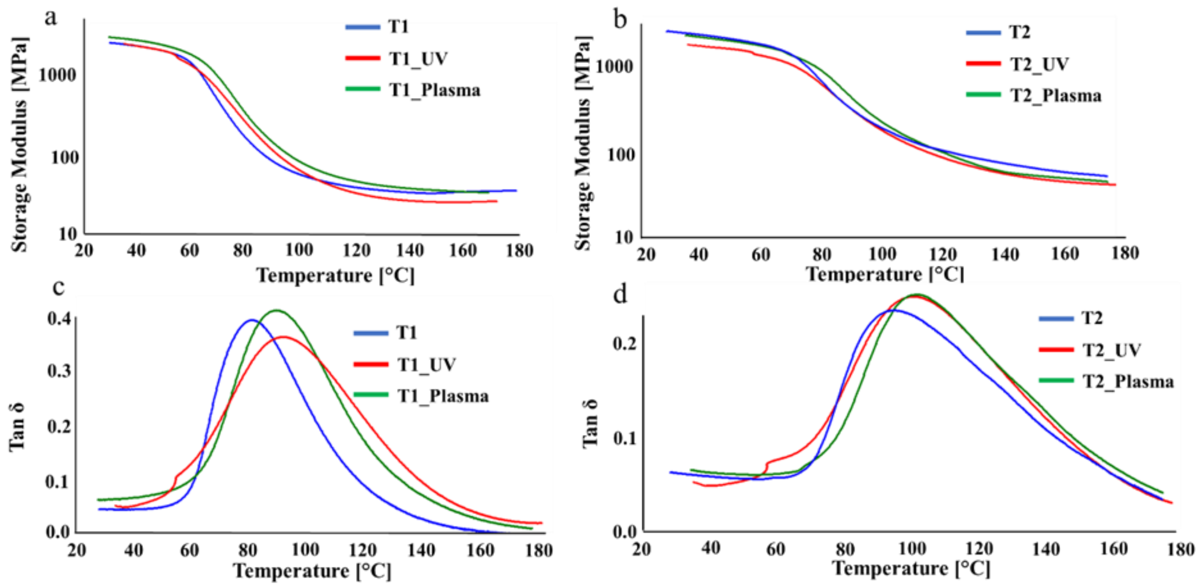

Figure S5. Storage moduli as a function of the temperature obtained from analysis in multifrequency strain mode on (a) T1, T1_UV, T1_Plasma and (b) T2, T2_UV, T2_Plasma; respective tan δ curves obtained on (c) T1, T1_UV, T1_Plasma and (d) T2, T2_UV, T2_Plasma.

Table S3. Weight increase of the thermosets after the lignosulfonate coating procedure.

**Sample**	**Weight increase after the coating procedure [w/w%]**
T1	0±0
T1_UV	0.8±0.4
T1_Plasma	2.7±0.3
T2	0.4±0.3
T2_UV	0.8±0.6
T2_Plasma	3.7±0.9

The test was performed in triplicate. Samples were weighted before and after the coating procedure. The weight increase was determined according with the following equation:

$$weight\ increase = \frac{w_f - w_i}{w_i} x100 \quad \text{(equation 2)}$$

Where w_f is the weight of the thermoset after the coating procedure and w_i is the initial weight of the thermoset.

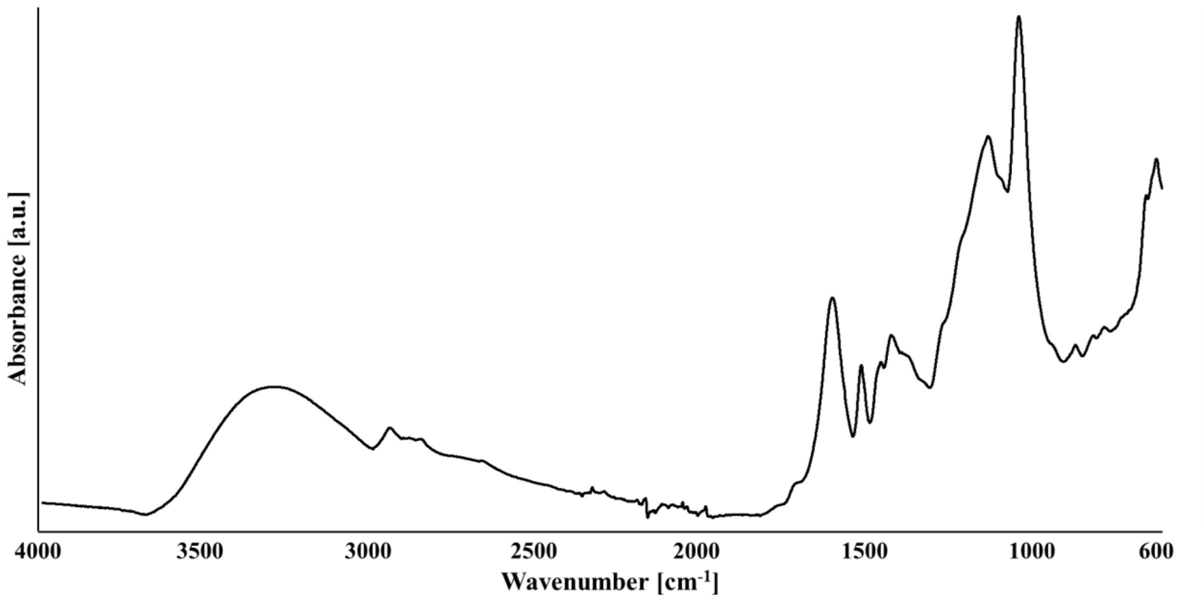

Figure S6. ATR-FTIR spectrum of lignosulfonate.

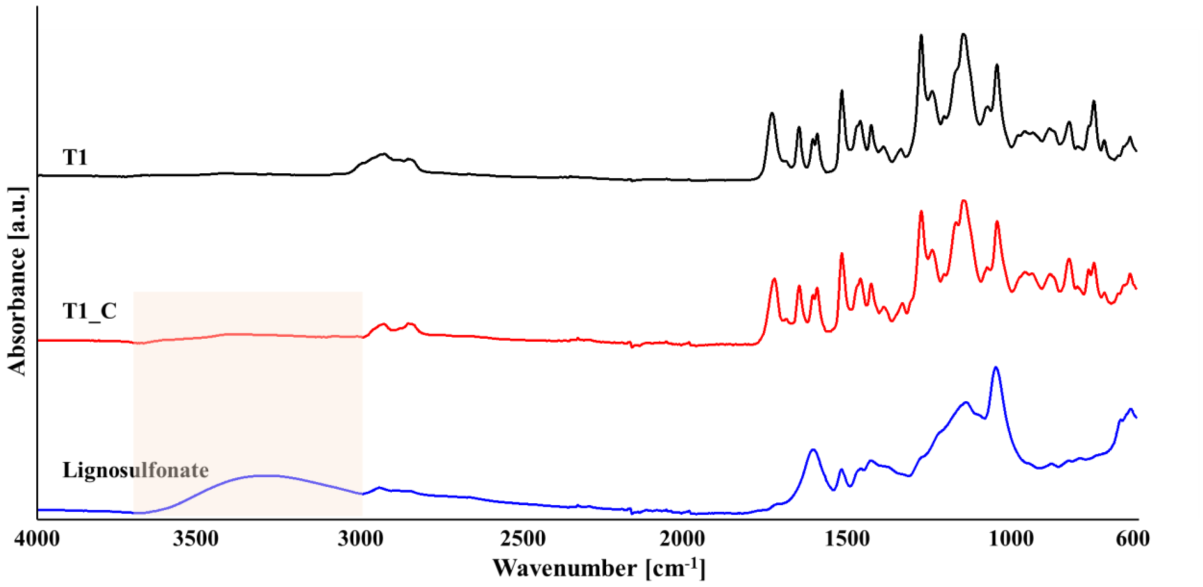

Figure S7. ATR-FTIR spectra of T1, T1_C and lignosulfonate.

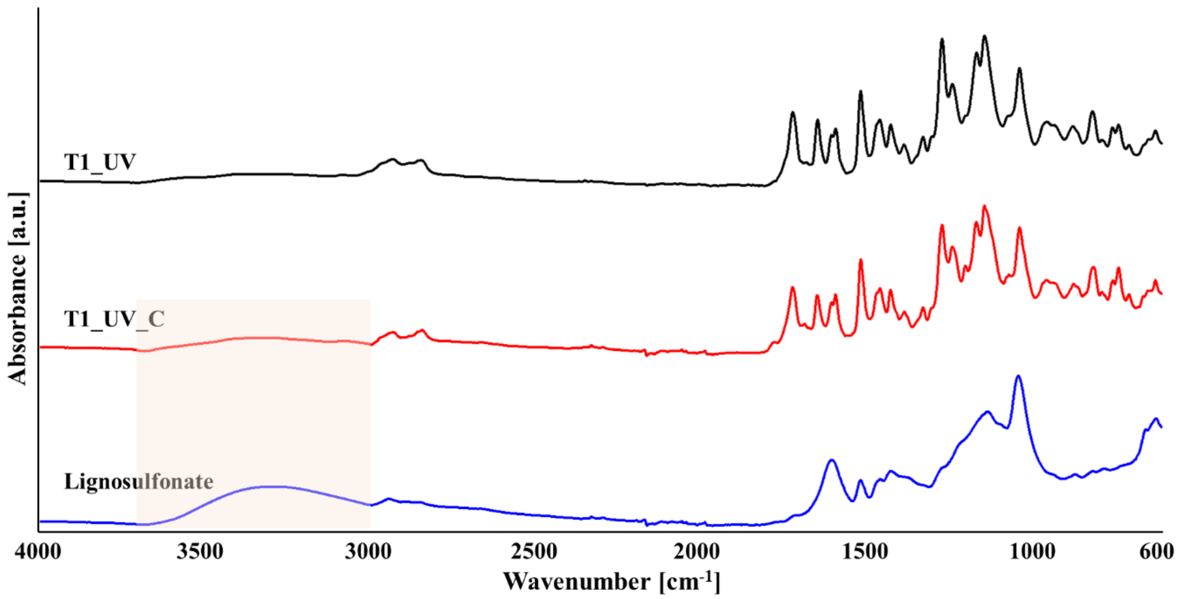

Figure S8. ATR-FTIR spectra of T1_UV, T1_UV_C and lignosulfonate. After the lignosulfonate coating procedure, an increase of the intensity in the region of the -OH absorption band can be observed on the surface of T1_UV_C.

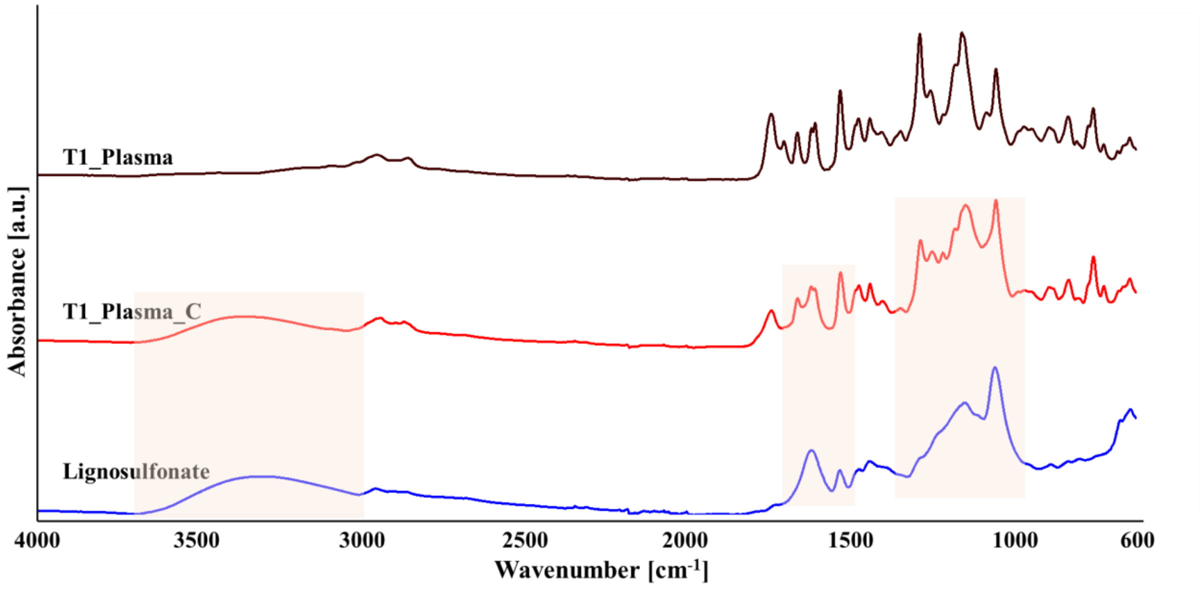

Figure S9. ATR-FTIR spectra of T1_Plasma, T1_Plasma_C and lignosulfonate. The presence of the lignosulfonate coating on the surface of T1_Plasma_C after the coating procedure is clearly confirmed.

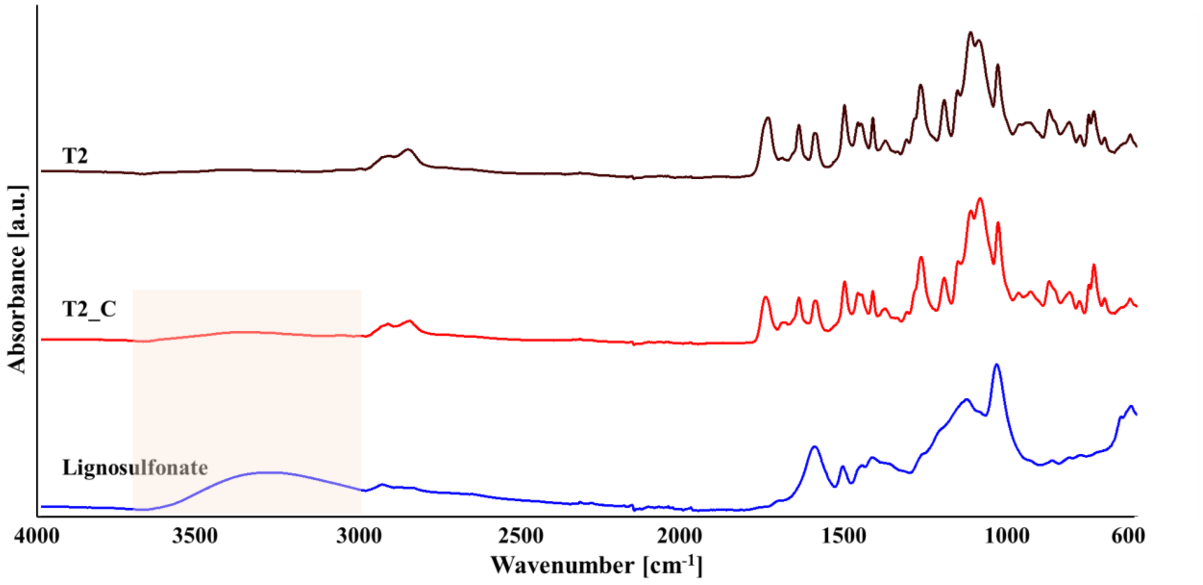

Figure S10. ATR-FTIR spectra of T2, T2_C and lignosulfonate.

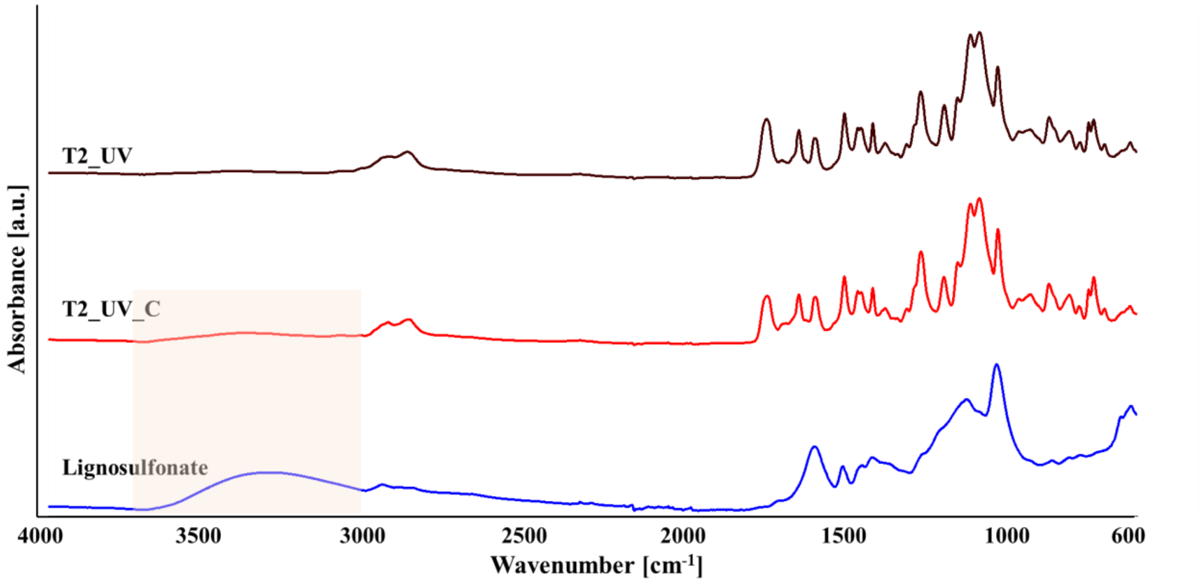

Figure S11. ATR-FTIR spectra of T2_UV, T2_UV_C and lignosulfonate. After the coating procedure, a slight increase of the intensity in the region of the -OH absorption band can be observed on the surface of T2_UV_C.

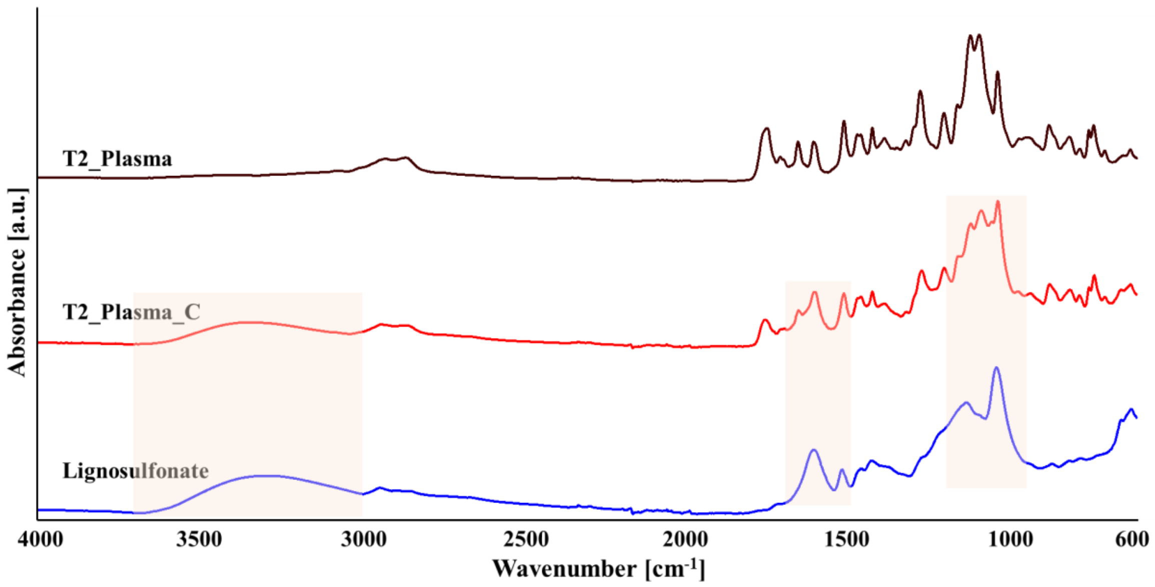

Figure S12. ATR-FTIR spectra of T2_Plasma, T2_Plasma_C and lignosulfonate. The presence of the lignosulfonate coating on the surface of T2_Plasma_C after the coating procedure is clearly confirmed.

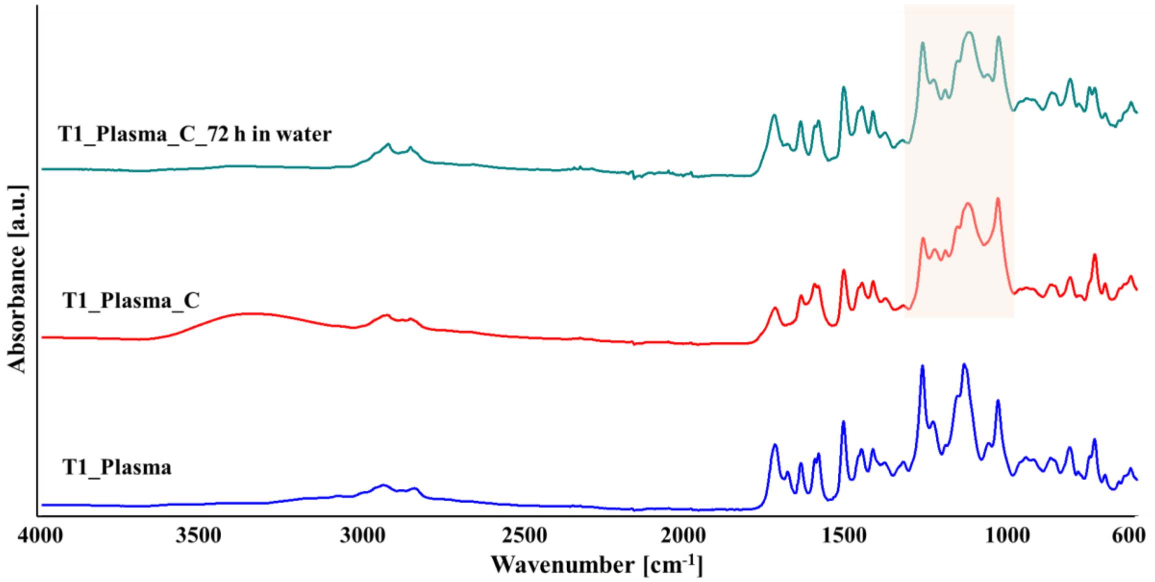

Figure S13. ATR-FTIR spectra of T1_Plasma_coated immersed in water for 72 h, T1_Plasma_coated and T1_Plasma. The presence of the coating on the surface of T1_Plasma_C can be detected also after 72 h immersion in water.

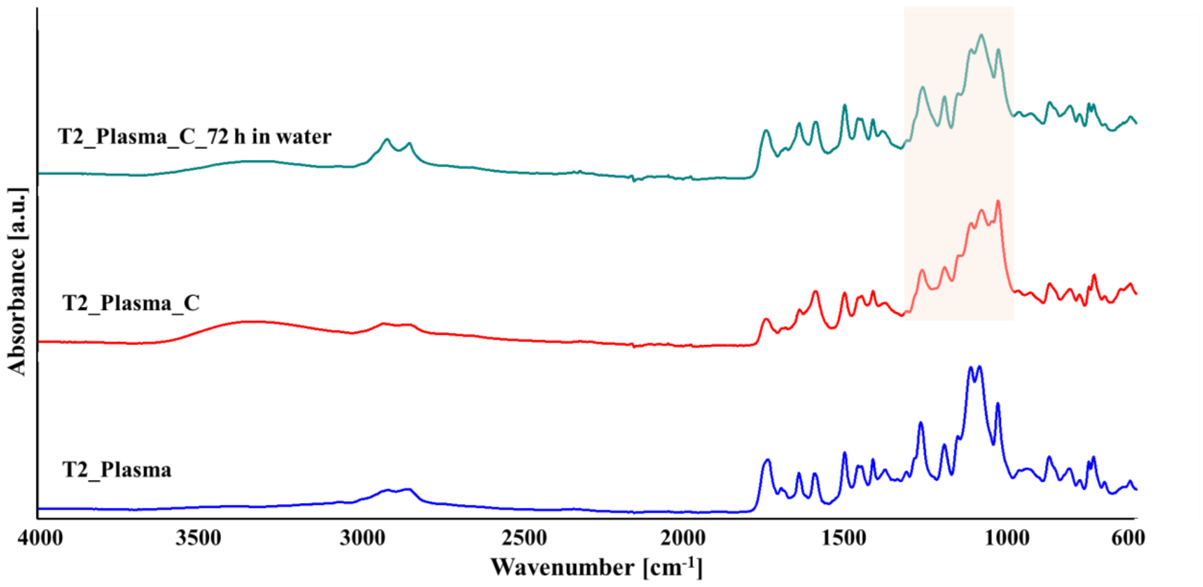

Figure S14. ATR-FTIR spectra of T2_Plasma_coated immersed in water for 72 h, T2_Plasma_C and T2_Plasma. The presence of the coating on the surface of T2_Plasma_C can be detected also after 72 h immersion in water.

Table S4. Residual DPPH° expressed as an average value with standard deviation for all the T1 samples without and with coating.

Sample	Time [min]	Residual DPPH° [%]
T1	10	20±5
	30	19±5
	60	9±3
	120	8±3
T1_UV	10	20±7
	30	16±2
	60	8±3
	120	6±1
T1_Plasma	10	20±6
	30	18±7
	60	9±3
	120	8±3
T1_C	10	14±3
	30	12±4
	60	10±2
	120	10±3
T1_UV_C	10	14±4
	30	10±0
	60	5±0
	120	0±0
T1_Plasma_C	10	11±3
	30	2±0
	60	0±0
	120	0±0

Table S5. Residual DPPH° expressed as an average value with standard deviations for all the T2 samples without and with coating.

Sample	Time [min]	Residual DPPH° [%]
T2	10	19±6
	30	12±6
	60	11±3
	120	13±6
T2_UV	10	23±8
	30	9±7
	60	11±5
	120	9±3
T2_Plasma	10	12±7
	30	10±3
	60	11±2
	120	12±6
T2_C	10	12±3
	30	5±0
	60	3±4
	120	1±1
T2_UV_C	10	9±1
	30	4±0
	60	3±0
	120	0±0
T2_Plasma_C	10	2±2
	30	1±0
	60	1±0
	120	1±0
